# Trade‐offs in avian parental care: a review of theory and meta‐analysis of brood size manipulations

**DOI:** 10.1002/brv.70181

**Published:** 2026-05-12

**Authors:** Rebekah A. McKinnon, Malgorzata Lagisz, Sheeraja Sridharan, David F. Westneat, Kimberley J. Mathot

**Affiliations:** ^1^ Department of Biological Sciences University of Alberta Edmonton AB T6G 2E9 Canada; ^2^ Ecology & Evolution Research Centre, School of Biological, Earth and Environmental Sciences The University of New South Wales Sydney NSW 2052 Australia; ^3^ Department of Biology University of Kentucky 101 Morgan Building Lexington KY 40506‐0225 USA

**Keywords:** life‐history trade‐offs, parental investment, provisioning behaviour, systematic review, theory, empirical tests

## Abstract

The selective forces shaping parental care have been studied for over 50 years. While theoretical and experimental work has yielded qualitative progress, the large body of empirical work testing predictions about parental investment based on life‐history trade‐offs has yet to be synthesized. We first provide an overview of the core life‐history theory exploring how selection might shape parental care. We then conduct a systematic review and meta‐analysis on studies that experimentally manipulated brood size in birds, a widely used experimental approach to manipulate parental investment. We extracted 313 estimates from 62 studies representing 31 species of birds from 19 different families and tested key predictions on trade‐offs in parental care derived from theory. Our analysis provides strong support for some predictions about life‐history trade‐offs in parental care, but weak or equivocal support for others. Specifically, we found that overall, avian parents respond to brood size manipulations as predicted by life‐history theory: they increased care in response to brood enlargement, and decreased care in response to brood reductions. Furthermore, for the same relative manipulation size, responses to brood reductions were greater than responses to brood enlargements. This finding is consistent with predictions derived from life‐history theory based on some types of non‐linear utility curves. However, many predictions derived from theory are not well supported by our comparative analysis. Species' life‐history traits such as clutch size (a measure of current reproduction), adult survival, and broods per year (two measures of future reproduction), explained little, if any, among‐species variation in response to brood size manipulations. Several factors may explain this. We highlight that brood size manipulations may affect more than just perception of the value of current reproduction, such as altering parents' perception of predation risk. Importantly, these unintended consequences could lead to asymmetric responses like those we observed. Other common experimental approaches – such as hormone manipulations, altering a partner's effort, and food supplementation – often affect multiple traits or fitness components simultaneously, or may involve cues that poorly match the evolved mechanisms guiding parental behaviour. Our review of both theory and experimental approaches suggests that there are multiple opportunities for more precise experiments. We offer several recommendations for effective designs. One is improved understanding of the biology underlying the functions relating to costs and benefits, with careful consideration of not only how the manipulation will affect only one of those, but also the mechanisms that might alter how parents perceive the manipulation. We also emphasize general principles, such as assessing alternative hypotheses and devising multiple independent tests. Armed with these recommendations, we believe there are new opportunities to increase the strength of inference achieved from studies aimed at understanding the trade‐offs affecting the evolution of parental care.

## I. INTRODUCTION

Parents in a variety of organisms provide resources to, or protection for, their offspring, and the level of parental care varies substantially among taxa (Balshine & Abate, 2021; Clutton‐Brock & Scott, 1991; Gilbert & Manica, 2010; Gubernick, 2013; Cockburn, 2006). The dominant explanation for this variation is that parental care is a life‐history trait subject to trade‐offs, primarily between current and future reproduction (Williams, 1966). Increasing parental care increases survival or quality of current offspring, but reduces one or more other components of parental fitness, such as the parent's ability to survive and reproduce in the future (Clutton‐Brock & Scott, 1991; Gross & Sargent, 1985; Trivers, 1972; Williams, 1966). The trade‐off between current and future reproduction is hypothesized to be influenced by the ecology of the organism (e.g. phenology of reproduction, food supply, or exposure to risks) and mating dynamics (Emlen & Oring, 1977), such that variation in ecology and mating systems leads to variation in parental care patterns. A central goal of research informed by this idea is to understand more precisely how parental care fits into general theory on life‐history trade‐offs.

There have been several challenges in attempting to evaluate the role of life‐history trade‐offs in shaping parental care decisions. Here, we first present a brief narrative review of existing theory that has developed predictions regarding the factors thought to influence how parental care becomes subject to life‐history trade‐offs. We then conduct a systematic review and formal meta‐analysis of studies implementing brood size manipulations in birds to test these predictions. The experimental nature of these studies allows us to evaluate the factors influencing life‐history trade‐offs at the within‐individual level (van Noordwijk & de Jong, 1986). We discuss the findings of our meta‐analysis and provide an overview of other types of empirical evidence currently available, identifying ideas with strong *versus* equivocal support. By evaluating current theory alongside presenting the results of a novel meta‐analysis of experimental work, we can assess the (mis)match between theory and empiricism and highlight important opportunities for furthering our understanding of parental care as a life‐history trait.

### (1) Theory

**Fig. 1:**
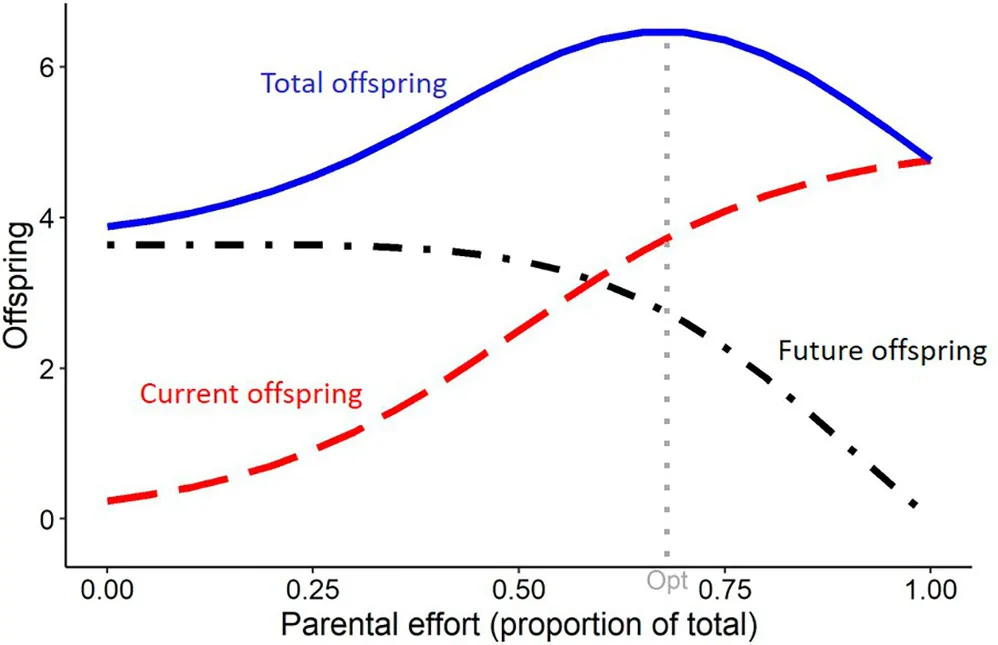
Graphical depiction of a life‐history model of parental care. Parental effort (scaled as a proportion of total effort available with pursuing mating or engaging in self‐maintenance comprising the other forms of effort) influences current offspring positively but non‐linearly (red dashed line) but reduces expected future offspring also called residual reproductive value, RRV), also non‐linearly. Expected future offspring is a function of extrinsic effects on parent survival and future reproduction but changes with parental effort due to its additional impact on gaining mates or surviving to a new breeding season. Total offspring (blue solid line) is the sum of current and future offspring, and the optimal level of effort is indicated by the vertical grey dotted line descending from the peak total number of offspring.

Theory on parental care is rooted in life‐history theory (Cole, 1954; Schaffer, 1974; Stearns, 1976; Gadgil & Bossert, 1970). Here we review extensions of the ideas, originally conveyed by graphical approaches, that capture continuous forms of parental care with effects on continuous fitness components (see Trivers, 1972; Chase, 1980; Gross & Sargent, 1985). These ideas were formalized by Winkler (1987) who modelled total reproductive value (lifetime reproductive success) split into current offspring and expected future offspring. All forms of parental care require effort, which is generally thought of as being in the form of time and/or energy. Increasing effort has a positive effect on current offspring but a negative effect on expected future offspring. When the relationships between effort and current offspring, and between effort and future offspring, are both linear, optimal levels of effort will either be 0% effort devoted to care, 100% effort devoted to care (presumably leading to semelparity), or implausibly, all values of effort leading to equal lifetime reproductive success. However, non‐linear functions (Fig. 1) produce an optimum where an intermediate value of effort maximizes the sum of current and future offspring (i.e. total offspring). The positive impact of effort on current offspring is defined as the benefit of care and the negative impact of effort on future offspring is defined as the cost of care. The optimal level of care occurs when the slopes of these two curves sum to 0, with lifetime reproductive success at a peak. This is illustrated graphically in Fig. 1 with some specific non‐linear functions relating effort to both current and future offspring.

Models of parental care have several key characteristics that influence predictions. First, to make the maths tractable, these models often assume continuous functions with relatively simple but non‐linear shapes. However, whether this assumed function is biologically relevant is often not known, which has important consequences for empirical studies designed to test models of optimal parental care. To illustrate, in Fig. 1, the function describing the relationship between effort and current reproduction (the benefit curve) is sigmoidal (Winkler & Wallin, 1987). This shape assumes that a change of 1 unit of effort has different effects on current offspring depending on how much effort is already allocated. This makes sense for some forms of care but not others. The sigmoidal shape is biologically reasonable for parental traits such as provisioning of offspring – a small increase in feeding when a parent is not providing much care will have little effect on offspring growth or survival (it is low regardless) and also little effect if the parent is providing lots of care already (it is high regardless due to a variety of constraints, such as limits on offspring digestive capacity and growth rate). Somewhere in between, a unit of care has a large impact on offspring fitness. Other forms of care, such as nest defence, might not produce this shape. Attendance at the nest may improve offspring survival *via* detection of potential predators (Rishworth & Pistorius, 2015; Riechert & Becker, 2017), but if the chances of a predator being encountered are constant through time, then increasing effort in the form of time at the nest will increase offspring survival linearly.

A second feature of many optimality models is the inclusion of interactions among variables that in turn influence the costs and benefits of parental care. Winkler (1987) built some of these interactions into his general model. For example, the impact of effort on current offspring depends on whether effort is shared among multiple offspring or not. If care is not shareable (such as provisioning of food, i.e. food consumed by one offspring cannot be consumed by another), then the shape of the benefit function depends on the number of offspring being cared for. Costs are also expected to differ depending on the age or condition of the parent (Webb *et al*., 2002) or other aspects of the life‐history of the species (Klug & Bonsall, 2010). Such interactions affect the predictions of the model and, if ignored, could alter the fit between test and theory.

Importantly, both the type of care and interactions with other variables can influence the fundamental shapes of some fitness curves. Since food provisioning is generally not shareable among offspring, the shape of this function depends on both effort and brood or litter size. The impact of brood size on this function is a key element of theory and yet is often forgotten when empirical tests such as brood size manipulations are used to test theory. While increasing brood size could naively be assumed to increase total care required, this is not necessarily the case. Some augmented brood sizes might actually reduce optimal care *via* the impact of brood size on the shape of the benefit curve, if the enlarged brood exceeds the size that can be supported in the current environment (Tammaru & Hõrak, 1999).

Some other issues with existing models have been recognized by theorists. Winkler's (1987) model and many of its extensions are static optimization models – they assume that all parameters are the same through time and among individuals and they do not incorporate how the behaviour of other individuals in a population might influence parameter values. By contrast, dynamic optimization models, which explore the ways in which feedbacks alter parameter values, generate individually unique optimal outputs that depend on population‐level attributes (Webb *et al*., 2002). Such models can produce intriguingly non‐intuitive outcomes. For example, Webb *et al*. (2002) modelled the effect of body condition and the behaviour of other individuals in the population on optimal parental care. Some costs, such as remating propensity of opposite‐sex individuals, may depend on the behaviour of others, and these can interact with other factors shaping parental care decisions. For example, more care may be predicted if there are few mating opportunities (within or across seasons) despite the energetic costs. By contrast, less care may be predicted when mating opportunities abound (within or across seasons), *and* energy affects the probability of future mating opportunities occurring. These theoretical approaches highlight several types of complexities, re‐emphasizing the role of interactive effects and individual differences in capabilities and condition. Many subsequent models have further developed potential interactions between variables. For instance, subareas in the study of parental care have arisen out of models on the role of partner care (Houston & Davies, 1985; Johnstone & Hinde, 2006; Kokko & Jennions, 2008), the influence of paternity on male care (Westneat & Sherman, 1993; Houston, 1995; Houston & McNamara, 2002), and the effects of additional mating opportunities (Emlen & Oring, 1977; Gross & Sargent, 1985; McNamara *et al*., 2000), with new theory incorporating more complex interactions and feedbacks (Alonzo, 2010; Araujo & Moura, 2023; Azad *et al*., 2022).

### (2) Factors predicted by life‐history theory to affect the parental response to brood size

Despite the complexities and context dependencies inherent to models of optimal parental care, several general predictions can be derived. If parental care is a life‐history trait subject to the trade‐off between current and future reproduction, then other aspects of life history may have major effects on the form and distribution of care both within and among species (Klug & Bonsall, 2010). For example, an increase in extrinsic mortality reduces residual reproductive value and therefore dilutes the potential negative impact of increased current care on future reproduction (all else being equal), thereby favouring the evolution of more extensive care. The same logic applies to terminal investment (Williams, 1966; Clutton‐Brock, 1984) whereby older individuals, potentially facing a higher probability of dying (actuarial senescence) before their next opportunity to reproduce, would be favoured to increase current reproduction despite its apparent cost. Thus, differences in mortality outside of reproduction (between species, between sexes, or between individuals of different qualities) would explain variation in parental care. Therefore, species with higher extrinsic mortality (lower adult survival probability) should increase care provided to current offspring more readily in response to brood size enlargements and decrease care less in response to brood size reductions compared to species with lower extrinsic mortality.

Other life‐history traits may also affect parental care in contingent ways. Age‐related changes in reproductive rate are one example. If reproductive potential declines beyond a certain age (i.e. reproductive senescence), this could favour increased investment in care by older individuals, as their lower potential for future reproduction reduces the potential long‐term costs associated with current care (Pianka & Parker, 1975). Another example is reproductive rate within a breeding season (Webb *et al*., 2002). Some species reproduce multiple times in a year, whereas others reproduce once a year or in some cases even once every other year. Reproductive rate may alter the costs of parenting and shifts in parental care can then feed back to alter reproductive rate (Alonzo, 2010; Araujo & Moura, 2023; Azad *et al*., 2022). Studies that examined the level of parental care provided to first broods have found no consequence on the frequency of individuals attempting a second brood (Evans Ogden & Stutchbury, 1996; Carro, Mermoz & Fernández, 2014; Cornell & Williams, 2016). However, individually optimized investment decisions in first broods may mask the costs of investment if, for example, higher quality individuals invest more in first broods and are more likely to breed again (*sensu* van Noordwijk & de Jong, 1986). By contrast, an experimental study found that providing food supplements to eastern bluebirds (*Sialia sialis*) during first broods increased the probability of females attempting second broods that season (O'Brien & Dawson, 2013). This suggests that the energetic cost of parenting the first brood in control nests is what prevented some females from renesting. Therefore, brood size manipulations in species with multiple breeding attempts per year should result in greater declines in care for reduced broods and smaller increases in care for enlarged broods, relative to species where only one breeding attempt per season is possible.

Across species, there is also marked variation in average clutch size, ranging from 1 to over 15, and smaller average clutch sizes correspond to more intensive parental care per offspring compared with larger clutch sizes (Bennett & Owens, 2002). Thus, species with naturally smaller clutch sizes are expected to have less ability to increase total effort with experimentally increased brood sizes. By contrast, given the lower *per‐capita* level of care in species with large clutch sizes, we predict they may maintain total effort when brood size is experimentally reduced as young are likely to benefit from the resultant increase in *per‐capita* investment. Specifically, larger natural clutch sizes should result in smaller declines in care in response to brood size reductions and greater increases in care in response to brood size enlargements.

Life‐history traits often co‐vary across species, with longer‐lived species generally having smaller clutches and fewer breeding attempts per year (Bennett & Owens, 2002). Given these patterns of covariation between life‐history traits related to current and future reproduction (Bennett & Owens, 2002), we predict that responses to brood size enlargements will decline with increasing lifetime productivity (i.e. total young produced), while responses to brood size reductions will increase.

### (3) Meta‐analysis of avian studies employing brood size manipulations

We conducted a systematic review and meta‐analysis of brood size manipulations in birds to test the predictions laid out above. Manipulations of brood size are the experimental approach of choice for manipulating levels of parental care in many taxa including insects (Ratz & Smiseth, 2018; Rauter & Moore, 2004), fish (Carlisle, 1985; Ridgway, 1989; Algera *et al*., 2017), and birds (Santos & Nakagawa, 2012; Drent & Daan, 1980), providing a large body of empirical work for synthesis. We focus our meta‐analysis on birds because they exhibit several useful characteristics for the purpose of testing predictions about life‐history traits in shaping the optimal levels of care. First, there is considerable variation in life history across avian species (Bennett & Owens, 2002), but all species are iteroparous to some degree. Second, parental care is common in birds yet shows considerable variation (Cockburn, 2006). Finally, the dominant form of care in birds is provisioning, which is relatively easy to measure.

To that end, we conducted a systematic review and meta‐analysis of brood size manipulations in birds to evaluate support for several predictions. Specifically, we predicted: (*i*) if current value does influence the level of care, then brood size manipulations should alter care with brood size reductions causing a decrease in care and brood size enlargements causing increased care; (*ii*) Asymmetries in the response (e.g. a larger absolute change in care to an experimentally reduced brood than to an experimentally enlarged brood) should be common as this is expected if fitness curves are non‐linear (see Fig. 1); (*iii*) The magnitude of the response to brood size manipulations should correlate with indices of life history. We considered the following life‐history traits: adult survival probability (a proxy for long‐term future reproductive opportunities); number of broods per year (a proxy for short‐term future reproductive opportunities); and clutch size (a measure of effort put into current reproduction). We predicted that species with higher annual survival, more breeding opportunities per year and/or smaller natural clutch sizes should exhibit smaller increases in care in response to enlarged broods, and larger decreases in care in response to reduced broods. Because these life‐history traits covary across species (Bennett & Owens, 2002), we also consider the effect of lifetime productivity, a composite trait that encompasses the combined effects of clutch size, broods per year, and survival, on responses to brood size manipulations at the species level; and (*iv*) Finally, because any experimental manipulation requires choices about the specific protocols used, we evaluated if there were effects of such methodological decisions on reported findings.

## II. METHODS

We report our systematic review and meta‐analysis process following the PRISMA‐EcoEvo guidelines outlined in O'Dea *et al*. (2021) (see online Supporting Information, Table S1).

### (1) Systematic literature search

**Fig. 2:**
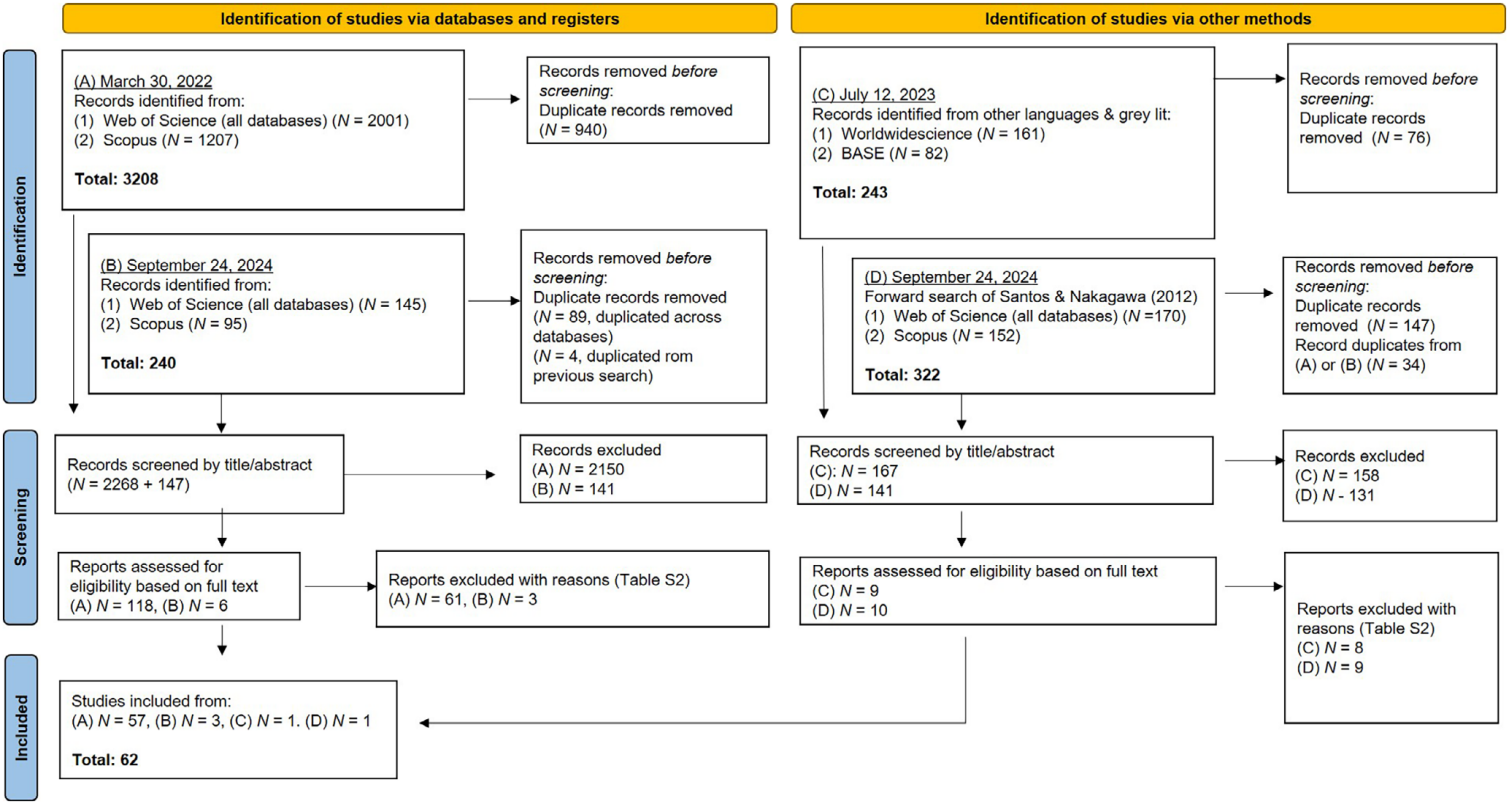
PRISMA flow chart showing the number of articles discovered and retained at each phase of the systematic review following four searches: (A) initial search of *Web of Science* and *Scopus* databases; (B) updated search of *Web of Science* and *Scopus* databases; (C) searches using *WorldWideScience* and *BASE* databases, and (D) forward searching of Santos & Nakagawa (2012). All included studies are identified in the reference list with an asterisk. All studies that were rejected after reading the full text are listed in Table S2, with reasons for exclusion.

We conducted searches in both *Web of Science* (Core Collection) and *Scopus* databases under the license of the University of Alberta. All available publication years were included (1927–2024), but results from these two databases were limited to articles written in English. We describe specific searches targeting non‐English language sources below. Each search was conducted using two groups of terms: one related to provisioning, and one related to brood demand. The two strings of search terms were combined using the ‘AND’ Boolean operator. Full search strings are provided in Appendix S1. Database searches were conducted on March 30th 2022 (initial search, ‘A’ in Fig. 2) and updated on September 24th 2024 (search ‘B’ in Fig. 2). The updated search was restricted to papers published in 2022 or later, to reduce duplication across the two searches.

We also conducted searches for articles written in languages other than English, and for studies from the grey literature (e.g. government reports, unpublished theses), using the same search strings outlined above. Searches were conducted using both *Worldwidescience* (https://worldwidescience.org/) and *Bielefeld Academic Search Engine* (*BASE*) (https://www.base-search.net/). *Worldwidescience* was a search engine maintained by the U.S. Department of Energy's Office of Scientific Information that enabled searches of national and international scientific databases to increase global representation in literature searches. As of April 2025, this search engine is no longer maintained; searches on this database thus can no longer be reproduced. However, we retain this search information here for completeness. *BASE* is a search engine maintained by the University of Bielefeld Library that enables multilingual searches, including databases for grey literature. Searches of *Worldwidescience* and *BASE* were conducted on July 12th 2023 (labelled ‘C’ in Fig. 2). We additionally identified potentially relevant studies by conducting a forward search of an earlier meta‐analysis examining the costs of parental care in terms of parental survival (Santos & Nakagawa, 2012). The forward search was carried out in both *Web of Science* and *Scopus* databases on September 24th 2024 (labelled ‘D’ in Fig. 2).

Following each of the searches described above, we first removed duplicates (e.g. when the same reference was discovered using different databases, or *via* earlier searches). After removal of duplicates, titles and abstracts were screened independently by two reviewers (R.A.M. and either K.J.M. or S.S.) using Rayyan (Ouzzani *et al*., 2016). When the two observers did not reach the same conclusion about inclusion or exclusion of an article, the title and abstract were discussed until consensus was reached. A total of 2,723 titles/abstracts were screened, from which we identified 143 studies to assess for inclusion based on their full texts (Fig. 2). Full texts were screened by R.A.M., and studies were selected for inclusion based on the following criteria. The study had to:

(i) Be conducted in a non‐cooperatively breeding bird species. We excluded cooperative breeding systems or systems with helpers at the nest because in such systems, costs of reproduction are spread across more individuals, influencing the trade‐off between current and future offspring.
(ii) Include an experimental manipulation of brood size (i.e. increase and/or decrease number of eggs or nestlings). Brood size manipulations must have been conducted using a random subset of nests in the population, or nests that reflected mean clutch/brood sizes. We excluded studies that conducted enlargements or reductions according to where along a gradient of natural brood sizes the nest fell, such as if only the smallest clutches received additional eggs and the largest clutches had eggs removed.
(iii) Include a control group. The control group could be nests that were either unmanipulated, or that experienced swapping of eggs or nestlings but no net change in number of eggs or nestlings. We additionally considered before/after designs as controls. For example, if provisioning behaviour was collected at the same nest both before and after the manipulation was carried out, the ‘before’ period was used as the control.
(iv) Provide one of the following measures of parental investment: feeding rate (i.e. provisioning visits per unit time), nest visit rate (i.e. if the observation protocol did not confirm presence of food on each nest visit), inter‐visit interval (i.e. time between two successive nest visits), or biomass delivery per unit time (i.e. incorporating both rate of food delivery and some measure of size/energy of delivered food items). Although other measures of provisioning behaviour are commonly reported (e.g. changes to diet breadth or food type delivered) these were not included as they could not be quantified for the purposes of a meta‐analysis or compared in a standardized way across different species. We did not consider forms of parental investment unrelated to provisioning (e.g. nest defence, faecal sac removal, or incubation). Additionally, we excluded studies that only provided estimates of parental care per nestling as opposed to at the level of the entire nest. This is because increased per nestling provisioning rate under brood size reduction could be consistent with either no change in total parental effort or decreased parental effort (and *vice versa* for brood enlargements), and our aim was to test predictions regarding total level of parental investment.
(v) Allow for extractions of estimates that did not include any additional manipulation(s) other than those described in point (ii) above that would change the cost of parental care. For example, estimates that included manipulation of offspring demand by food supplementing or by starving provisioning adults or nestlings, applying weights, or handicapping adults were excluded as these manipulations add an additional constraint on provisioning behaviour. In some cases, studies that included additional manipulations reported results for control groups that experienced brood size manipulations but no additional interventions; these estimates were extracted and included in the meta‐analysis.
(vi) Provide extractable information that allowed us to calculate an effect size (Hedges' *g*) from either descriptive or inferential statistics, sample size, and a measure of uncertainty. This information could be extractable from the article, supplementary material, accompanying data sets, extracted from figures, or obtained directly from the authors. To allow estimation of uncertainty, comparisons that had less than *N* = 3 nests for any treatment level required to calculate treatment effects were excluded.

A total of 62 studies were identified for inclusion (indicated with an asterisk in the reference list). All texts that were excluded with reasons (*N* = 81) were additionally reviewed by K.J.M. to ensure they did not meet eligibility criteria. These excluded studies are listed in Table S2 along with their reasons for exclusion, and the database search from which they were discovered. The full PRISMA flow chart is provided in Fig. 2.

### (2) Data extraction and effect size calculation

Detailed descriptions and definitions of all data extracted from each study are provided in Table S3, but in brief, these included: the stage at which the treatment was applied (eggs or nestlings); the duration of the manipulation treatment, which was later categorized as either long (>1 day) or short duration (<1 day). We additionally noted several other details from each study, as outlined in Table S3. We did not assess the quality and methods of data collection within studies (e.g. whether behavioural observations were made using nest cameras or by live observation).

If an effect size was not directly provided within the study results, we extracted descriptive statistics over inferential statistics. We recorded the mean, SD and SE for all eligible measures of parental investment. When means and SDs or SEs were not provided in the main text but were instead illustrated in figures, we used WebPlotDigitizer version 4.5 (Rohatgi, 2022) to extract data from figures (accessible at https://automeris.io/WebPlotDigitizer.html). Where only one of SE or SD was provided, we calculated the other using the equation: SE = SD/√*N*, where *N* = number of nests. We used this to calculate Hedges' *g* effect sizes using the ‘escalc’ function from the ‘metafor’ package in R version 4.4.1 (R Core Team, 2024). We calculated Hedges' *g* as our standardized effect size because several studies included in our meta‐analysis involved small sample sizes, and Hedges' *g* is better suited to deal with small study sample sizes than other standardized effects sizes such as Cohen's *d* (Hedges & Olkin, 2014). Data were extracted by R.A.M. and a subset of extractions (15 out of 62 studies) were reviewed by K.J.M. to ensure consistency and accuracy.

### (3) Data wrangling and data coding

We predicted that, at the level of the nest, parental effort would increase in brood enlargement treatments and decrease in brood reduction treatments. However, depending on the response variable, increased parental effort could lead to an increase in the measured response variable (e.g. provisioning rate, such as deliveries per hour), or decrease in the response variable (e.g. the time between consecutive provisioning visits, inter‐visit intervals, IVIs). Consequently, we introduced an additional column (‘ES_flip’) to our datasheet, which was subsequently used to adjust the sign of Hedges' *g* values in each corresponding row. The resultant column ensured that a positive effect size consistently indicated a change in the response variable in the predicted direction based on theory; increased effort for brood size enlargements, and decreased effort for brood size reduction. For example, for enlarged broods, an increase in provisioning rate, increase in delivery rate, or decrease in IVIs would be coded as positive. By contrast, for reduced broods, a decrease in provisioning rate, decrease in delivery rate, or increase in IVI would be coded as positive because these would be changes in the predicted direction. This adjusted effect size was used in subsequent analyses. In the interpretation of our findings, a higher positive value of effect sizes denotes a more pronounced behavioural change in the predicted direction. This coding allowed us to compare the absolute magnitude of response to brood size enlargement *versus* brood size reduction, despite anticipated responses being in opposite directions on the observed scale.

We were also interested in evaluating whether there was a phylogenetic effect on responses to brood size manipulations. Given that there are some unresolved branches in the avian phylogenetic tree, we linked our data set to two different phylogenetic trees to verify that any observed effect (or lack of effect) of phylogeny was robust to differences between phylogenies. The two phylogenetic trees used were obtained from Open Tree of Life (available at https://tree.opentreeoflife.org/opentree/argus/opentree14.9@ott93302) and from Jetz *et al*. (2012), respectively. We created and compared separate meta‐analytic models each using the phylogenetic information generated by the different trees.

### (4) Life‐history data

Since we were interested in the influence of residual reproductive value (RRV) on provisioning response to brood size manipulations, we obtained estimates of the following life‐history parameters from the literature for each of the species represented in the 62 studies included in our quantitative review: annual adult survival probability, age at first reproduction, maximum species longevity, mean clutch size, and mean number of broods per year. These data were obtained from the study itself, when available, or from ‘Birds of the World’ by Cornell (accessible at https://birdsoftheworld.org/bow/home), or the British Trust for Ornithology (https://www.bto.org/our-science/data). A detailed description of the prioritization of sources used to obtain different life‐history variables is provided in Appendix S2, and the sources of life‐history trait values used in the meta‐analysis for each species are provided in Table S4.

### (5) Meta‐analyses

We conducted all statistical analysis in the program R version 4.4.1 (R Core Team, 2024) using the RStudio interface Version 2024.04.2 (R Studio Team, 2024). We implemented our statistical models using the *rma.mv* function from the ‘metafor’ package as this is suitable for conducting phylogenetic multi‐level meta‐analysis and meta‐regressions (Viechtbauer, 2010). For all models, we included the Hedges' *g* value as our response variable (observed effect size) and the inverse of sampling variances of the response variable as weights. We subsequently calculated the multi‐level equivalent of heterogeneity (*I* ^2^), which quantifies variance not attributable to sampling error, for both total heterogeneity (*I* ^2^_[total]_) and for each random effect (Nakagawa & Santos, 2012; Higgins & Thompson, 2002). *I* ^2^ is presented as a percentage (%) and can be used to determine if heterogeneity is high (*I* ^2^ ~ 75%), moderate (*I* ^2^ ~ 50%), or low (*I* ^2^ ~ 25%) (Nakagawa & Santos, 2012).

We created meta‐analytic models in three steps. First, we assessed which random effects to include in the models. To do this, we ran null meta‐regression models with no predictor variables but each of the following random effects: phylogeny (the phylogenetic effect of species), species ID (the non‐phylogenetic effect of species), study ID (unique value for each study), and observation‐level ID (i.e. a random effect with a unique level for each estimated effect size). We ran two separate null meta‐regressions; one for each phylogenetic tree (see Section II.3). For all of the models described below, we retained the following random effects which were identified as being important as they explained >1% of the variance in estimated effect sizes (see Section III): study ID, observation‐level ID, and the phylogenetic effect of species. Analyses presented in the main text include the phylogenetic effect of species based on the phylogeny from Jetz *et al*. (2012), but results for all models presented were qualitatively and quantitatively similar using the Tree of Life phylogeny (see Tables S5–S7).

Using meta‐regression (mixed‐effects models), we first assessed support for an effect of treatment (brood enlargement *versus* brood reduction), and whether the treatment effects were moderated by life‐history traits. To do this, we modelled Hedges' *g* as a function of treatment using the final random effect structure as described above. First, we included only treatment (enlarged or reduced) as a moderator. Subsequently, we included each of the life‐history traits outlined above (adult survival, broods per year, clutch size, lifetime productivity) both as main effects, and in interaction with treatment, as we explicitly predicted that these would have asymmetric effects on responses to brood size enlargements *versus* brood size reductions.

Next, we evaluated the importance of six methodological moderators. (*i*) Magnitude of the brood size manipulation (calculated as number of eggs/nestlings added/removed divided by natural clutch size/brood size). The magnitude of the response should be sensitive to the change in the relative value of care; depending on the shapes of the costs and benefits curves, parental care may increase linearly with brood size, or peak at intermediate brood sizes (Tammaru & Hõrak, 1999). Therefore, we evaluated support for both linear (effect size of brood size manipulation equal for brood size reductions and brood size enlargements) and non‐linear effects of manipulation magnitude on parental effort (i.e. whether the effect sizes of brood size manipulations on parental effort were different for brood size reductions *versus* brood size enlargements); (*ii*) Treatment stage (whether researchers moved eggs or nestlings to create enlarged or reduced broods); (*iii*) Treatment duration [whether the study was short term (≤1 day) or long term (>1 day)], which could affect response magnitude if parents respond to moment‐to‐moment variation in brood demand differently than to long‐term variation in brood demand; (*iv*) Response variable category (3 levels: visit rate, feeding rate, biomass delivery rate); (*v*) Study design [whether the experiment involved a within‐subject control (i.e. before–after study design), or among‐subject control]; (*vi*) Parental sex, to test whether there was evidence that response strength differed between males and females (and both parents together). For the last five variables, we did not have *a priori* predictions about the effect direction. These analyses revealed that some, but not all, methodological moderators affected the strength of response to brood size manipulations (see Section III). Therefore, we visually evaluated whether any of the methodological moderators were confounded with treatment, or with variables of key interest such that they might influence our inference regarding key life‐history traits, which they were not (see Fig. S1).

For all models, we assessed the importance of moderators by calculating marginal *R* ^2^ (reported as percentages) following recommendations by Nakagawa & Schielzeth (2013). We interpret moderators with a marginal *R* ^2^ of ~1% as having a small effect, marginal *R* ^2^ ~ 6% as having a medium effect, and marginal *R* ^2^ of 14% or greater as having a large effect, following Cohen (2013). Parameters whose 95% CI did not overlap zero are interpreted as statistically significant, whereas parameters whose estimates were biased away from zero, but whose 95% CI overlapped zero, are referred to as showing a trend or tendency. When drawing comparisons between pairs of estimates, we considered them statistically different when the proportion overlap of a single arm of each 95% CI was ≤0.50, as this corresponds to a *P* value of approximately 0.05 (Cumming, 2009). To visualize meta‐analytic results, we used the R packages ‘ggplot2’ (Wilkinson, 2016), ‘orchaRd’ (Nakagawa *et al*., 2023), ‘ggaluvial’ (Brunson & Read, 2019) and ‘metafor’ (Viechtbauer & Viechtbauer, 2015).

### (6) Publication bias and sensitivity analyses

We evaluated evidence for publication bias using funnel plot asymmetry in addition to testing the significance of the asymmetry using a multilevel version of Egger's regression (Nakagawa *et al*., 2022). We calculated the harmonic mean of sample sizes for each study (‘effectN’) and from this included ‘effective N’, i.e. the square root of ‘effectN’, as a fixed effect in our Egger's regression model as well as random effects for study ID, observation‐level ID, and the phylogenetic effect of species. Calculating the harmonic mean of sample sizes is a strategy to address potential bias introduced by studies with very small or very large sample sizes.

We also evaluated the sensitivity of our results to three extreme estimates that were identified both by visual inspection of the funnel plots and inspection of the distribution of Hedges' *g* values. These outlier estimates were all extracted from the same paper (Study ID 60; Ardia, 2007). We therefore performed sensitivity analysis by removing this study (*k* = 8 estimates total) from our data set and comparing the results of our final models with *versus* without this study included.

Finally, we assessed the presence of a time lag effect by regressing our standardized effect sizes against publication year, i.e. ‘decline effect’ (Yang, Lagisz & Nakagawa, 2022) again including random effects for study ID, observation‐level ID, and the phylogenetic effect of species. Time‐lag effects occur when studies reporting significant results are more likely to be published early compared to studies reporting non‐significant results, leading to a decline in effect size over time (Yang *et al*., 2022; Jennions & Møller, 2002; Sánchez‐Tójar *et al*., 2018).

## III. RESULTS

### (1) Overview

**Fig. 3:**
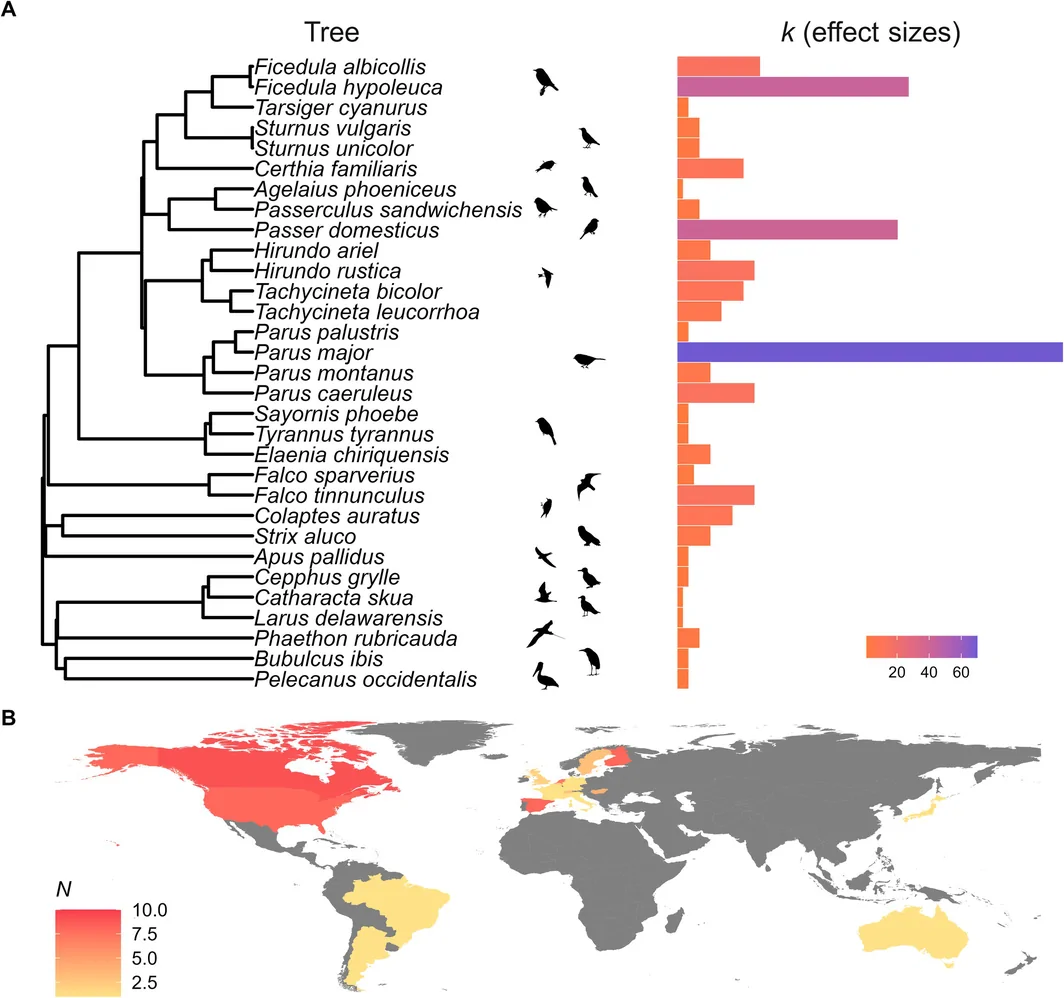
Illustration of phylogenetic and geographic breadth of estimates included in the meta‐analysis. (A) The phylogenetic relationships (left) used in the meta‐regression presented in the main text (Jetz *et al*., 2012), and the number of effect sizes (*k*) available for each species. Silhouette images depict the 19 avian families included in the data set. (B) The geographic distribution of studies. The colour of the country on the gradient from yellow to red represents the total number of studies (*N*) included in the meta‐analysis that were conducted in that country. Silhouettes representing different avian families were obtained under Attribution‐ShareAlike 4.0 International license from phylopic.org. Detailed copywrite information for all images can be accessed at: https://www.phylopic.org/permalinks/8914c2418cf8444921070dd0205828ae75ad20e8f8223adc0343b1b09466462b.

We extracted a total of 313 estimated effects sizes (*k*) from 62 studies that conducted brood size enlargements (*k* = 151) and/or brood size reductions (*k* = 162). Estimates were obtained for 31 species of birds (Fig. 3A). The three most common species in the dataset were the great tit (*Parus major*) with *k* = 70 estimates from *N* = 13 studies, pied flycatcher (*Ficedula hypoleuca*) with *k* = 42 estimates from *N* = 7 studies, and house sparrow (*Passer domesticus*) with *k* = 40 estimates from *N* = 2 studies. Estimates were obtained from studies in 19 countries/territories, though these were primarily from Europe (40 studies across 11 countries) and North America (17 studies across two countries). Only one study came from Asia (Japan), two from Oceania (Australia, Christmas Island/Johnstone Atoll), and two from South America (Brazil and Argentina), with none from either Africa or Antarctica (Fig. 3B). The most frequently reported response variables were feeding rate (*k* = 213 estimates) followed by nest visit rate (as a proxy for feeding rate; *k* = 73 estimates) and biomass delivery per unit time (*k* = 27 estimates).

Our meta‐analysis also included species with a wide range of average annual adult survival probability, from 0.30/year for the fairy martin (*Hirundo ariel*) to 0.90/year for great skuas (*Catharacta skua*) and red‐tailed tropicbirds (*Phaethon rubricauda*). Overall average annual adult survival was 0.60. Average number of broods per year ranged from 1 to 2, with an overall mean of 1.4. Our meta‐analysis included species with a wide range of mean natural clutch sizes (mean: 6.3, range: 1–9). Although there was no correlation between clutch size and broods per year (*r* = 0.08, 95% CI = −0.25, 0.39, *t* = 0.46, *P* = 0.65) in our data set, adult survival probability and clutch size were significantly negatively correlated across species/populations (*r* = −0.51, 95% CI = −0.72, −0.22, *t* = −3.54, *P* = 0.0011), as were adult survival probability and broods per year (*r* = −0.41, 95% CI = −0.65, −0.10, *t* = −2.66, *P* = 0.012). Therefore, we calculated lifetime productivity (LRS) as a composite life‐history trait to capture potential additive and/or synergistic effects across these correlated life‐history traits on parental care decisions as follows:

$$\mathrm{LRS}=\begin{array}{l} \sum_{i=f}^{L}(\text{(Clutch size)}\times \left(\text{Broods}\;\mathrm{per}\;\text{year}\right)\\ \times {\text{(Adult survival probability)}}^{i-1}) \end{array}$$

where *f* = age at first reproduction, and *L* is maximum reported longevity.

### (2) Random‐effects‐only meta‐analytic models

Our null models revealed important contributions of observation‐level ID, study‐level ID, and the phylogenetic effect of species in contributing to total heterogeneity. The observed total heterogeneity was moderate (*I* ^2^_[Total]_ ~ 50%), implying that responses to brood size manipulations were contingent on additional moderators; this set the stage for the meta‐regression models described below. For these meta‐regression models, we retained study ID (*I* ^2^_[Total]_ ~ 40%), estimate ID (*I* ^2^_[EstimateID]_ ~ 5%), and the phylogenetic effect of species(*I* ^2^_[Phylogeny]_ ~ 1.5–5%), as these were identified as important sources of variation in our random‐effects only models (Table S5). The non‐phylogenetic effect of species was not an important source of variation (*I* ^2^_[SpeciesID]_ < 0.001%), and therefore, was not included in subsequent meta‐regressions. Results were similar whether using the phylogeny from Tree of Life or Jetz *et al*. (2012) (see Table S5). Results presented in main text use the phylogeny obtained from Jetz *et al*. (2012).

### (3) Meta‐regression models with moderators

First, we tested our three main biological predictions that (*i*) parents would respond to brood size manipulations by increasing (for enlargements) or decreasing (for reductions) care, (*ii*) that responses would be larger for brood reductions compared to brood enlargements, and (*iii*) that asymmetric responses to enlargements *versus* reductions would be mediated by life‐history traits associated with variation in levels of current *versus* future reproduction. As predicted, type of brood size manipulation explained a small amount of the total variation in provisioning response (*R* ^2^_[marginal]_ = 1.97); responses to experimental brood reductions (ß = 0.47, 95% CI = 0.28, 0.66) were stronger than responses to experimental brood enlargements (ß = 0.36, 95% CI = 0.18, 0.54), and this difference was statistically significant (estimated difference ß = 0.11, 95% CI = 0.01, 0.21) (Tables S6 and S7).

**Fig. 4:**
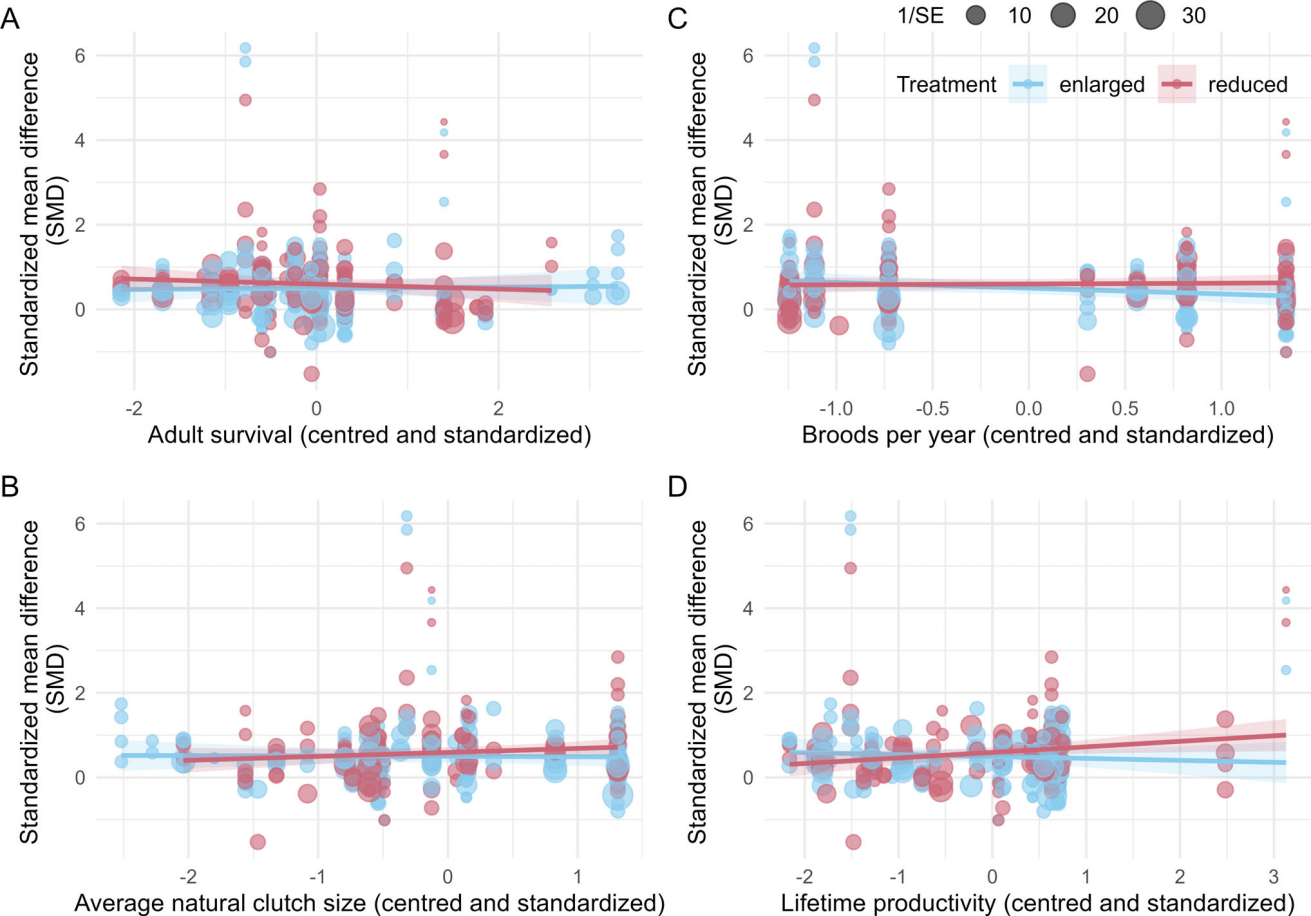
Estimated effects of (A) adult survival probability, (B) clutch size, (C) number of broods per year, and (D) lifetime productivity, on responses to brood size enlargements (blue, *k* = 151 estimated effect sizes) and brood size reductions (red, *k* = 162 estimated effect sizes). Size of the points represents 1/SE, so that more precise estimates (lower SE) are represented with larger points.

Support for manipulation‐related differences in response to brood size manipulations as a function of life‐history traits was generally weak (Fig. 4). Adult survival probability (Fig. 4A) and clutch size (Fig. 4B) had little explanatory power (*R* ^2^_[marginal]_ = 2.42 and *R* ^2^_[marginal]_ = 2.19, respectively) above that of the type of brood size manipulations alone (*R* ^2^_[marginal]_ = 1.97), providing no support for our initial predictions. Manipulation‐specific effects of number of broods per year (Fig. 4C) and lifetime productivity (Fig. 4D) had some explanatory power above that of manipulation type alone (*R* ^2^_[marginal]_ = 3.54 and *R* ^2^_[marginal]_ = 4.81, respectively). There was moderate support that the effect of the number of broods per year differed for brood enlargements *versus* brood reductions (difference: ß = 0.10, 95% CI = −0.01, 0.20). Specifically, species which produced more broods per year tended to have smaller responses to brood size enlargements (ß = −0.06, 95% CI = −0.21, 0.09), but not brood size reductions (ß = 0.04, 95% CI = −0.11, 0.18) (Fig. 4C), providing mixed support for our predictions. There was also moderate support that the effect of lifetime productivity differed for brood enlargements *versus* brood reductions (difference: ß = 0.05, 95% CI = −0.06, 0.16). Specifically, species with higher lifetime productivity tended to respond more strongly to brood reductions (ß = 0.08, 95% CI = −0.05, 0.22) than to brood enlargements (ß = 0.03, 95% CI = −0.11, 0.17) (Fig. 4D).

Total heterogeneity was similar across all models that included life‐history moderators (*I* ^2^_[Total]_ range: 47.94–50.54), indicating that important sources of heterogeneity in response to brood size manipulations were not captured by the life‐history traits considered.

**Fig. 5:**
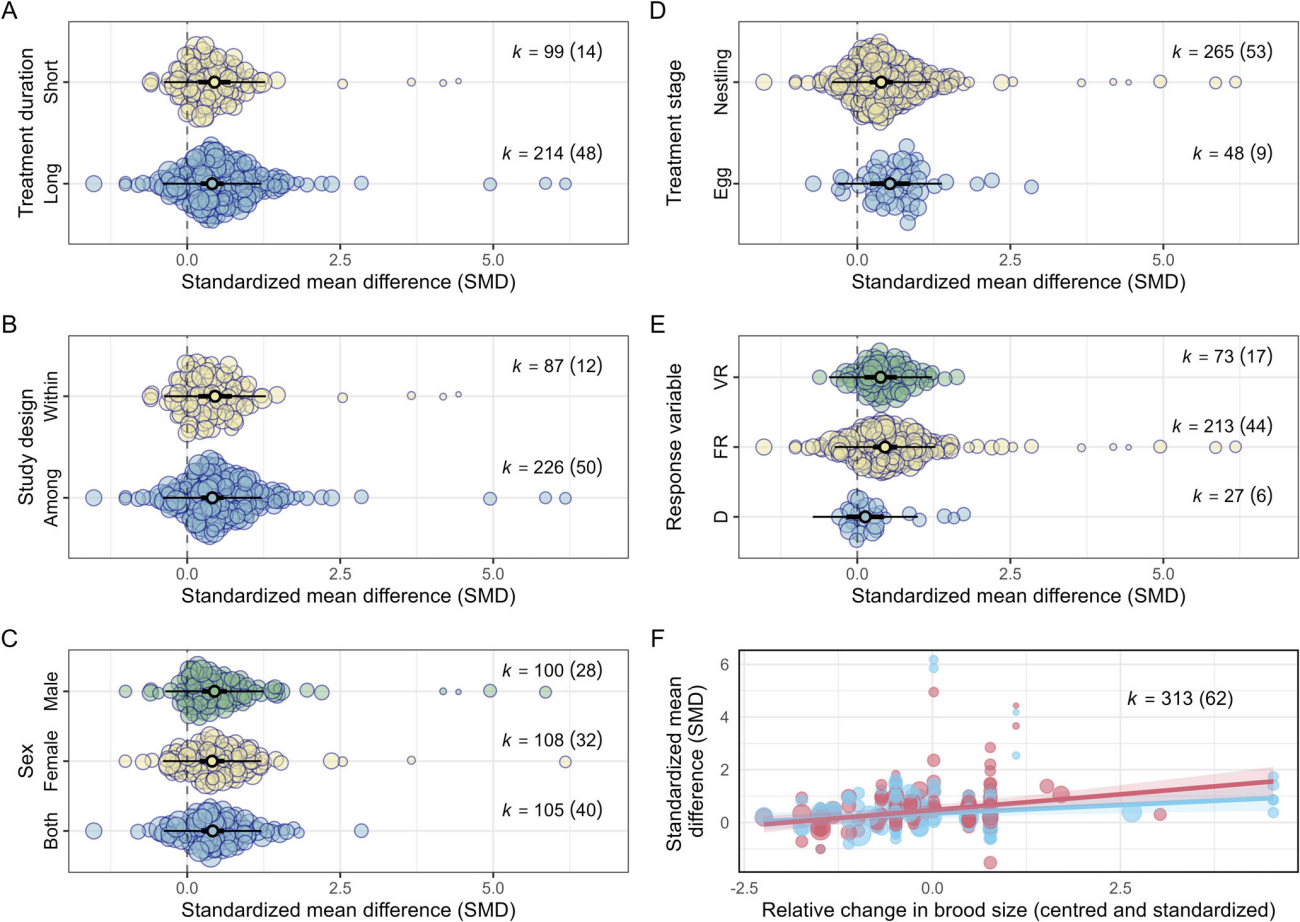
Illustration of the estimated effect of different methodological modifiers (*y*‐axis) on the response to brood size manipulations (*x*‐axis). (A) Treatment duration category (short = within a day, long = more than 1 day), (B) study design (within‐ or among‐subject), (C) focal parent sex (male, female or both sexes combined), (D) treatment stage (eggs or nestlings), (E) response variable category (D = delivery, FR = feeding rate, or VR = visit rate), and (F) relative change in brood size (axes switched) *via* manipulation (centred and standardized). For all panels, the size of the coloured dots is inversely related to the SE of the estimate so that larger dots indicate estimates with higher precision. In A–E, the black circles denote the meta‐analytic means, the black rectangles represent the 95% confidence intervals, and whiskers denote the 95% prediction intervals. In F, blue dots represent estimates from brood size enlargements, and red dots represent estimates from brood size reductions.

Finally, we ran a series of meta‐analytic regressions to investigate the importance of several potential methodological moderators of the response to brood size manipulations. We found that treatment duration (two levels: short = within 1 day or long = greater than 1 day) (*R* ^2^_[marginal]_ = 0.19) (Fig. 5A), study design (two levels: within‐subject or among‐subject design; *R* ^2^_[marginal]_ = 0.24) (Fig. 5B) and focal parent sex (three levels: both sexes combined, male, female; *R* ^2^_[marginal]_ = 0.17) (Fig. 5C) did not explain variation in response to brood size manipulations.

A small to moderate amount of variation in response to brood size manipulations was explained by treatment stage (*R* ^2^_[marginal]_ = 1.66) (Fig. 5D) and response variable category (*R* ^2^_[marginal]_ = 4.74) (Fig. 5E). Manipulations that occurred at the nestling stage (ß = 0.39, 95% CI = 0.20, 0.58) tended to produce smaller responses than manipulations conducted at the egg stage (ß = 0.53, 95% CI = 0.20, 0.86), although the difference was not significant (ß = −0.14, 95% CI = −0.45, 0.17). The magnitude of response to brood size manipulations was largest when the response variable was feeding rates (ß = 0.45, 95% CI = 0.26, 0.65), followed by visit rates (ß = 0.38, 95% CI = 0.12, 0.65). Effects on delivery rates tended to be positive, but were not different from zero (ß = 0.13, 95% CI = −0.18, 0.44).

By contrast, the magnitude of the brood size manipulation (expressed as relative change, centred and standardized) explained substantial variation in the strength of response (*R* ^2^_[marginal]_ = 18.10). Specifically, larger manipulations produced larger parental responses in the predicted direction (ß = 0.18, 95% CI = 0.09, 0.27). We also evaluated whether the effect of relative magnitude varied as a function of manipulation type (reduction *versus* enlargement). There was a small increase in explained variation when modelling relative manipulation strength in interactions with treatment (*R* ^2^_[marginal]_ = 20.89). Overall, the effect of relative manipulation strength was approximately twofold greater in response to brood reductions (*β* = 0.24, 95% CI = 0.13, 0.35) compared to brood enlargements (*β* = 0.13, 95% CI = 0.03, 0.23), and this difference (estimated difference: *β* = 0.11, 95% CI = 0.00, 0.23) was significant (Fig. 5F).

### (4) Sensitivity analysis and publication bias

**Fig. 6:**
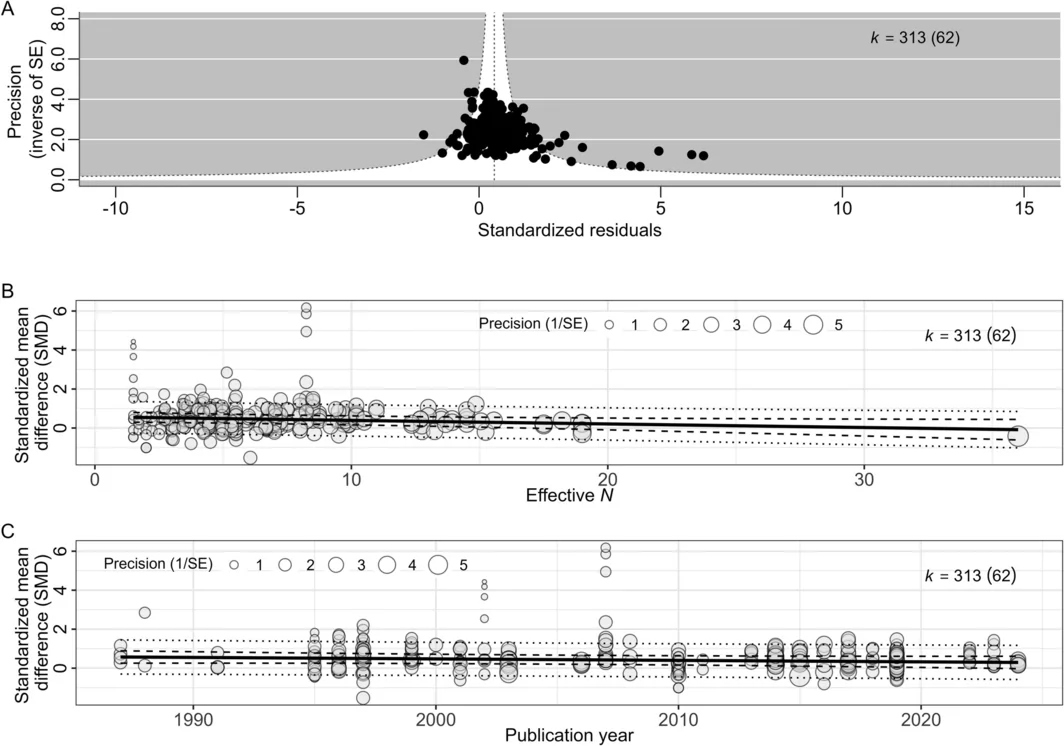
Publication bias assessments. (A) A funnel plot of effect sizes plotted against their precision calculated as 1/SE. Note that the three outlier data points on the right side of the funnel are from Ardia (2007) (see Table S8). (B) Plot illustrating the small but significant relationships between effective sample size and standardized mean difference. (C) Time lag bias plot showing the lack of relationship between publication year and standardized mean difference.

Visual assessment of the funnel plot did not reveal asymmetry in estimated effect sizes, which would provide evidence of publication bias (Fig. 6A), although the three right‐most estimates on the funnel plot were identified as potential outliers. These three estimates all came from the same study (Ardia, 2007) and had Hedges' *g* values markedly higher than other estimates in our data set (i.e. Hedges' *g* values of >4.9 where the 4th highest value was 4.4; see Table S8). Therefore, we excluded all estimates taken from that study (*k* = 8), and reran all analyses described in the main text. Results with *versus* without this study were qualitatively similar (see Tables S5–S7). Total heterogeneity in the null models declined when this study was excluded, but the amount of heterogeneity explained by each of the considered random effects was similar to analyses with the full data set (Table S5). Similarly, conclusions about important methodological and life‐history moderators were largely unchanged (Table S6), although reported effects were generally stronger for the analysis using the reduced data set (Tables S6 and S7), presumably due to reduced unexplained variance. We highlight in the discussion any instances where alternative data sets generated qualitatively different results to those based on the full data and the Jetz *et al*. (2012) phylogeny.

Egger's tests showed a small but significant effect of effective sample size (*N*) on standardized mean difference (SMD) in effect size (*β* = −0.02; 95% CI = −0.04, 0.00; *P* = 0.034; Fig. 6B). Larger estimated effect sizes tended to be associated with smaller sample sizes, suggesting that small studies reporting significant results were more likely to be published, however, the estimated effect size was small, suggesting the bias was relatively low. There was no evidence for a shift in effect sizes over time (*β* = −0.01, 95% CI = −0.02, 0.00; *P* = 0.20; Fig. 6C).

## IV. DISCUSSION

Our meta‐analysis provides several important insights into how the experimental approach of manipulating the value of current care *via* brood size serves to assess general theory about parental care. Overall, brood size manipulations altered parental care patterns in predicted directions; increasing the number of offspring generally increased care and reducing the number of offspring reduced care, providing quantitative support for the qualitative impression gained from individual studies. Furthermore, we found strong support for an interaction between the relative size of the manipulation and manipulation direction. Larger relative manipulations generated stronger responses, and response strength to brood reductions was approximately double the response strength to brood enlargements of the same relative magnitude. Although life‐history traits including clutch size, broods per year, adult survival and lifetime productivity explained some variation in response to brood size manipulations, these effects were modest, and moderate heterogeneity in effect sizes remained even after accounting for these life‐history traits. Some of this variation was due to methodological decisions, yet methodology may interface with some aspects of the biology of care to provide new insights, which we examine in more detail below.

### (1) Are responses to brood size manipulations mediated by life‐history traits?

Our results provide modest support for the idea that aspects of biology that influence the costs of care (e.g. Winkler, 1987) shape parental care decisions (Fig. 4). We found asymmetry in provisioning response to brood enlargements *versus* brood reductions with parents responding more strongly to brood reductions compared to brood enlargements. Further, the degree of this asymmetry was higher in species which produced more broods per year (Fig. 4C) and for species with higher lifetime reproduction (Fig. 4D). Asymmetry in response to brood enlargements *versus* brood reductions is consistent with the idea that a non‐linear cost curve is more extreme if more future reproduction is at stake (Fig. 1). Such asymmetries also ought to be affected by how manipulations alter the shape of the benefit curve (Tammaru & Hõrak, 1999), yet this likely depends on the precise shapes of both curves, which we cannot infer from the data we have collected. However, we note that these effects explained only a small (all *R* ^2^_[marginal]_ < 5% when using complete data set) to moderate (*R* ^2^_[marginal]_: ~7–14% when excluding outliers from Ardia (2007), see Table S6) amount of variation, suggesting that additional factors are important.

What those additional factors are is not clear. Other metrics predicted to influence parental responses, such as adult survival and clutch size, had almost no explanatory value (*R* ^2^_[marginal]_ ~ 2% when using the complete data set, *R* ^2^_[marginal]_: ~5% when using the reduced data set), providing little support for theory invoking these variables (Klug & Bonsall, 2010; Klug, Bonsall & Alonzo, 2013). There are two potential reasons for this. One is that adult survival and clutch size are typically negatively correlated (Bennett & Owens, 2002), and this means that brood size manipulations are necessarily scaled differently in long‐lived species. For example, the two longest lived species in our data set, the red‐tailed tropicbird (maximum reported age = 32 years) and great skuas (maximum reported age = 38 years), have mean natural clutch sizes of 1 and 1.5, respectively. Thus, brood size enlargements are necessarily relatively large in these species because adding one egg or nestling doubles, or nearly doubles, brood size, but also, brood reductions are not possible.

A second issue in evaluating the importance of life history in shaping parental care decisions is that there may be several confounding explanations. For example, a species may be long‐lived because they avoid costly care, or because care is not costly. The estimates of species‐specific adult annual survival probability used in the present study combine multiple sources of mortality, making it impossible to tease apart these explanations. One issue is whether changes in care within a species cause changes in RRV. In an earlier meta‐analysis, Santos & Nakagawa (2012) compiled 29 studies on 19 species of free‐living birds and found mixed evidence that manipulated brood size affected adult survival. However, Santos & Nakagawa (2012) found only *N* = 11 studies that simultaneously measured changes in care and changes in adult survival (a proxy for RRV), which prevents meaningful analysis linking the strength of changes in parental care to the strength of changes in RRV. A more recent meta‐analysis of the effect of brood size manipulations on adult survival found strong support that experimentally increasing clutch size decreased adult survival in an analysis of *N* = 39 studies of *N* = 28 species (Winder, Simons & Burke, 2025), suggesting that inducing increased care *via* manipulations of brood size does reduce RRV.

### (2) Are responses to brood size manipulations mediated by methodological decisions?

We also found no support for sex‐related differences in response to brood size manipulations; response strength was quantitatively similar whether responses were documented in males, females, or pooled across sexes (Fig. 5C). This contrasts with the sex‐specific effects on survival reported by Santos & Nakagawa (2012). They found that males caring for enlarged broods had significantly lower survival (~25%) than control males, but no effect of caring for enlarged broods was observed in females. Moreover, reductions in brood size appeared to have little effect on survival of either sex. However, a more recent meta‐analysis based on a larger sample of species found no evidence for sex‐related differences in the survival consequences of brood size enlargements (Winder *et al*., 2025). Unfortunately, the effects of brood size reductions on parent survival were not investigated by Winder *et al*. (2025). Nonetheless, their finding that males and females pay similar survival costs when tending to experimentally enlarged broods are consistent with our observation of no sex‐related differences in response to brood size manipulations.

We evaluated the importance of several other methodological decisions in affecting response strength to brood size manipulations. There was no support for differences in response strength as a function of treatment duration. Response magnitudes were quantitatively similar whether manipulations were short‐term (i.e. ≤1 day) or long‐term (i.e. >1 day) (Fig. 5A). Many birds adjust their behaviour in response to begging intensity (Wright & Leonard, 2002), which likely reflects nestling hunger or condition (Mock, Dugas & Strickler, 2011). Adding or subtracting nestlings from a brood alters overall begging intensity, and for parents that assess this at the nest level, this may explain why they respond to brood size enlargements potentially as quickly as after a single visit to the enlarged brood (Westneat & Mutzel, 2019). If, however, parents attend to the begging intensity of individual nestlings, then we would expect parental responses to occur only after some delay following the change in size as each nestling in an augmented brood would get hungrier over time and begin to beg more strenuously. Our finding that responses to short‐term *versus* long‐term manipulations were quantitatively similar suggests that attending to nest‐level begging may be the more common means by which parental effort is determined.

There was also no support for differences in response strength for studies that adopted a within‐subject (e.g. before–after) *versus* among‐subject (e.g. control *versus* manipulation groups) design (Fig. 5B). This is reassuring, since random assignment of treatment categories to different nests should capture within‐subject effects. Similarly, there was no support for an effect of timing of manipulation (i.e. egg stage or nestling stage) on parental response strength (Fig. 5D). This suggests that parents do not make a sealed bid for parental investment based on the number of eggs that were laid or number of young that hatched (*sensu* McNamara, Gasson & Houston, 1999). Sealed bids can vary in their timing, but if the assessment occurs at hatching, then we would have expected manipulations at the egg stage to generate stronger responses compared to manipulations at the nestling (post‐hatching) stage.

We found that the strength of response to brood size manipulations was associated with the category of the response variable recorded; feeding rate and visit rate were strongly affected by brood size manipulations, but delivery rate was not (Fig. 5E, Tables S6 and S7). This suggests that increases in feeding/visit rates to the nest may be partially facilitated by bringing smaller prey, such that higher feeding/visit rates do not scale linearly with rates of biomass delivery, although variation in this scaling exists (Mathot *et al*., 2017; Westneat & Mutzel, 2019). This highlights that parental responses to changes in brood demand are multivariate; any single proxy of parental effort may fail to capture overall levels of parental effort if different parental responses offset one another. For example, increased visit rates in response to brood enlargements may overestimate the effect of brood enlargements on parental effort if the increase in visit rate is achieved by reducing search effort and accepting lower quality prey (Wright *et al*., 1998), which may simultaneously change only slightly the cost of such effort.

### (3) Limitations

Our meta‐analysis provides strong support for several predictions derived from parental care theory. Namely, we found that parents adjust levels of care in response to current brood size, that responses to brood enlargements are smaller than responses to brood reductions, and that responses to brood enlargements tend to be smaller in species where more future reproduction is at stake. However, our meta‐analysis provided weak or equivocal support for the effects of other life‐history traits, with most of the heterogeneity in effect sizes being explained by study ID. This suggests that responses to brood size manipulations are context specific, and that there are important factors shaping responses which were not captured in our analysis. The available estimates also come from a small number of species relative to the total number of bird species (*N* = 31 out of >10,000), with almost 50% of estimates coming from just three species (Fig. 3A). This limited our ability to tease apart phylogenetic effects from life‐history effects. Further, over 90% of included studies were conducted in Europe or North America (57 of 62 studies) (Fig. 3B). This may have limited our power to detect effects of life‐history traits that vary geographically (Bennett & Owens, 2002).

Another limitation is that the complex ecology of parental care complicates interpretation in several ways. For example, effect sizes were larger when brood size was reduced than when it was enlarged. While this is consistent with theory involving non‐linear cost curves (see above and Fig. 1), one seldom‐acknowledged issue with brood size manipulations (but see Westneat & Mutzel, 2019) is that while natural decreases in brood size – through partial predation (Martin *et al*., 2000) or brood reduction (Lack, 1947) – are common in many bird species, natural increases in the number of *nestlings* within a breeding attempt never occur. This asymmetry raises questions about why parents respond to experimental brood enlargements at all and suggests that responses to experimental enlargements and reductions may be shaped by different ecological and evolutionary expectations. Brood size reductions might interface with the normal begging mechanisms used to adjust care in addition to cues that are unique to reductions. For instance, if partial predation occurs naturally, it may predict two unique issues for parents. First, if the predator is a danger to parents as well as nestlings (Ghalambor & Martin, 2001), then additional risks may be incurred when returning to a recently reduced brood. Second, the value of the remaining nestlings may be reduced below their numerical value if the predator is likely to return. These issues differ in the underlying impact of the manipulation on variables invoked by theory. In option 1, the manipulation is changing the perception of risk to the parent, making it about costs of care and not value of care. In option 2, the value of care is shifted but in ways that differ from a brood size enlargement since it is not about food provided by a parent but the impact of an external source of mortality on the offspring. Some populations may be more sensitive to brood size reductions, complicating interpretation through a life‐history lens. However, how brood size manipulations interface with natural mechanisms for adjusting care has rarely been considered in experimental design or interpretation.

### (4) Can other experimental approaches provide different insights?

The results from our meta‐analysis provide at best modest support for predictions derived from life‐history theory. We suggest this may be in part because brood size manipulations alone are insufficient to test life‐history theory rigorously as it relates to parental care decisions. Researchers have manipulated avian parental care in other ways, including hormone manipulations, handicapping, and food supplementation. Below, we discuss whether these three common alternative approaches can effectively reveal costs of care.

First, we know of five studies that have manipulated levels of the male steroid hormone testosterone, and tracked effects on parental care to assess the hypothesis that testosterone mediates shifts between parental care and either territory defence or pursuit of additional mating opportunities (Ketterson *et al*., 1992; McGlothlin, Jawor & Ketterson, 2007; Raouf *et al*., 1997; van Duyse, Pinxten & Eens, 2000; George, Navarro & Rosvall, 2021). These studies have documented shifts in parental care (Ketterson *et al*., 1992; McGlothlin *et al*., 2007; van Duyse *et al*., 2000; George *et al*., 2021), courtship behaviour (Ketterson *et al*., 1992), and paternity (Raouf *et al*., 1997). Thus, while hormone manipulations have potential to reveal costs of parental care, the effects of testosterone on life‐history decisions are multivariate (Ketterson & Nolan, 1992), and no study has measured all the relevant components simultaneously. Moreover, too few species have been studied to assess aspects of theory such as whether other life‐history traits impact the trade‐offs affecting care.

A second type of experimental approach has focused on species with biparental care, in which researchers manipulate the care of one parent downward, by removing one parent entirely or by handicapping them, either by adding weights or clipping flight feathers (reviewed in Harrison *et al*., 2009). Removal of a partner is expected to shift the remaining parent to lower on the benefit curve (to the left in Fig. 1), while the latter two are both expected to increase the cost per unit of care provided. Most of these experiments were not designed to investigate costs of parental care but were focused on testing aspects of theory about how partners split the total care provided to offspring (Houston & Davies, 1985). It is generally inferred that weighting or clipping increases the costs of care and Harrison *et al*. (2009) found widespread support for handicapping reducing the care of the target parent, with variation among specific techniques (weights had a bigger impact than clipping). However, studies that have investigated the consequences of handicapping have uncovered a variety of other impacts including on sexual behaviour (Griggio *et al*., 2008), parental body mass (Leclaire *et al*., 2011), metabolic capacity (Fowler & Williams, 2017), oxidative stress (Fowler & Williams, 2017), nest failure rates (Hegemann *et al*., 2013), and parental immune response (Fowler & Williams, 2017; Hegemann *et al*., 2013). As with hormone manipulations, these results indicate that altering the effort individuals must exert to provide parental care clearly has costs, but handicapping may affect multiple traits simultaneously, reducing their ability to test specific components of life‐history theory about parental care. Additionally, given that removal or handicapping decreases care provided by the target individual, this has the presumed impact of increasing the value of the care of the other parent. Indeed, over a suite of studies, the partner of handicapped or removed parents increased their care, although there was significant heterogeneity among those cases (Harrison *et al*., 2009), and some more recent studies using the same technique in parallel on five species did not find this response (Wagner, Mourocq & Griesser, 2019). As far as we know, no studies have assessed the consequences of the trickle‐down effects of handicapping on partner fitness.

Food supplementation is a third approach for manipulating aspects of parental care. Conceptually, providing food changes the ecological conditions under which care is expressed and has the potential to address directly the general idea of ecology altering components of trade‐offs and explaining diversity in care patterns. A strength of this approach is that altering food resources could fit well into the evolved mechanisms of parental decision‐making. Food supplements have been used in a variety of species to explore the impact of environmental conditions on timing of breeding (Drent & Daan, 1980; Ruffino *et al*., 2014), clutch size (Ruffino *et al*., 2014), and nestling growth (Ruffino *et al*., 2014). Unfortunately, as for the handicapping studies, many have been focused on goals other than assessing life‐history theory. For example, most studies seek to confirm hypotheses about key components of habitats explaining differences in breeding performance among years or sites (Ruffino *et al*., 2014). In some studies, food supplements to parents assessed the idea that food constraints may cause parents to lose condition or otherwise limit their parenting (Cucco & Malacarne, 1995; Récapet *et al*., 2016).

In one notable exception, Markman, Pinshow & Wright (2002) separated food provided to offspring from food used by parents in Palestine sunbirds (*Cinnyris osea*). They found that supplemented parents shifted time budgets from self‐feeding to offspring feeding (females) or nest defence (males), experimentally demonstrating that if per‐unit costs to parents (supplements make self‐feeding more efficient per unit time) are reduced, care can increase. Moreover, Markman *et al*. (2002) found that nestlings of parents fed the higher quality diet, which was never fed to the offspring, put on more mass than controls. This is an incisive manipulation that supports the trade‐off idea, although we note that further progress could be gained by manipulating nestling food and adult food in both directions and tracking how food resources influence fitness of both adults and young. Food supplements were also used to assess costs of brood care in a species of fish, the smallmouth bass (*Micropterus dolomieu*), in which offspring are guarded but not provisioned (Zolderdo *et al*., 2016). While supplements did not alter the frequency of parental behaviour, they significantly reduced nest abandonment, supporting the idea that brood care is energetically costly and that cost was reduced by supplemented food. This result suggests a step function for costs of parental care, rather than a continuous curve (see Fig. S2), since supplementation affected the binary decision to care or not, rather than a decision to shift the amount of care. The examples of sunbirds and bass illustrate the potential of food supplementation for assessing life‐history theory about parental behaviour. More studies that can pinpoint different components of the trade‐off and, ideally, manipulate them in both directions, would advance the testing of theory significantly.

### (5) Future directions

Brood size manipulations, hormonal manipulations, manipulations of levels of care provided by partners, and food supplementation share similar important limitations when applying them to tests of parental care theory. Here, we outline approaches that could be adopted in future studies to begin to address these limitations.

The first limitation is that each of these approaches may alter traits besides parental behaviour, either directly or indirectly. For example, experimentally increased testosterone levels may directly increase investment in courtship behaviour or increases in courtship may arise secondarily due to a testosterone‐mediated decrease in parental care. Determining the extent to which effects are direct *versus* indirect is critical for evaluating support for life‐history trade‐offs, and may provide insights into evolved mechanisms for allocating time or resources to trade‐offs among activities. Future studies should assess whether suites of behaviours share a common underlying mechanism (e.g. testosterone levels) or exhibit correlated changes due to trade‐offs, by measuring multiple traits simultaneously and applying methods like structural equation modelling to quantify direct and indirect relationships (Araya‐Ajoy & Dingemanse, 2014).

A second limitation is that manipulation aimed at testing trade‐offs between current *versus* future reproduction may alter more than one component of fitness. Handicapping, for instance, appears to increase the energetic cost per unit of parental effort, but may also reduce the relative benefits of alternative activities – such as seeking mates, self‐foraging, or predator avoidance – thereby lowering the opportunity costs of providing care. Similarly, providing supplemental food may be useful to either the parent or the offspring and thus may influence more than one variable in the trade‐off simultaneously (McKinnon *et al*., 2024). Ideally, experimental manipulations testing a trade‐off should be targeting only the per‐unit effects on one component. However, in the absence of methods that can manipulate a single component of the trade‐off, future studies can still achieve stronger inference about the importance of isolated components by testing multiple hypotheses, and devising multiple tests (e.g. using multiple manipulation methods) of the same hypothesis (e.g. Platt, 1964).

Finally, most manipulations assume open‐ended plasticity by parents – manipulated cues are assumed to be the same as natural cues used by evolved mechanisms to manage parental care. As we noted above, experimental brood reductions may affect parental perception of both costs and benefits of parental care. Other forms of manipulation may also suffer from this general issue. There have been few attempts to assess whether a given manipulation results in a mismatch between the cues that were altered and those the subject has evolved to employ. This is a specific case of the more general problem of leveraging a hypothesized mechanism while testing hypotheses about function (Tinbergen, 1963). Future studies would benefit from carefully considering mechanisms of assessment by parents before designing an experiment to test functional hypotheses (McNamara & Houston, 2009).

An overarching theme in our review of experimental studies is that empirical work would benefit from being more tightly tied to mathematical theory. Most studies rely on verbal descriptions of the trade‐offs, yet modellers have repeatedly shown that predictions depend on the exact shapes of cost–benefit curves (e.g. Houston, 2012). Consider a system that might be appropriate for testing the model depicted graphically in Fig. 1. While the cost and benefit curves seem plausible, effectively manipulating them requires more information. For example, why might costs accelerate? If the trade‐off is over time allocation, and the cost of parenting is taking time away from self‐maintenance (i.e. self = 1 – parental effort), then one possibility is that parent self‐maintenance has a similar diminishing returns curve to the offspring curve; at very low self‐maintenance, adding a bit more has large effects on parental condition, but at higher levels of self‐maintenance, that increase has a smaller relative impact on parental condition. Thus, Markman *et al*.'s (2002) manipulation of the efficiency at which a parent can self‐feed in Palestine sunbirds effectively manipulated the cost curve and not the benefit curve. This was possible because adult sunbirds feed on a different resource for themselves than the food they supply to dependent offspring and the two types require different foraging (Markman, Pinshow & Wright, 1999).

In other species, such as European starlings (*Sturnus vulgaris*), parent food overlaps with offspring food and is found in similar places (Wright *et al*., 1998; Wright & Cuthill, 1990), minimizing the time allocation trade‐off. In such species, cost curves may nevertheless accelerate because foraging exposes parents to predators. At low levels of parental effort, parents could focus their foraging in safe places with little effect on RRV, but as offspring demand increases, parents may face an additional trade‐off: remain safe but provide inadequate food or shift to foraging also in less‐safe locations. If they do the latter, then the impact on RRV would accelerate as that shift increases. Manipulations of these costs could involve manipulating food resources (ideally in both directions) in the safer places, which would require knowing where those are. Another implication of this possibility for the biology of a cost curve is that the actual cost is mortality of the parent that occurs during the period of parental care. Most of the papers we used in our meta‐analysis and in the Santos & Nakagawa (2012) meta‐analysis did not state whether mortality during parental care occurred during the brood manipulations, or if it did, how such cases were treated. We suspect many researchers might have omitted such broods from the analysis because the focus is often on delayed costs rather than immediate ones, and the loss of a parent affects the measurement of parental response and the duration of the treatment. However, this is unfortunate because clearly predation is a potential force affecting life‐history (Martin, 1995). A deeper understanding of the causes of cost curve shapes might have forestalled such decisions.

The supplementation study in smallmouth bass (Zolderdo *et al*., 2016) illustrates another biological underpinning to a cost curve. In many fish, body condition may be traded off against care (van den Berghe, 1992). Thus, costs do not accumulate per unit of specific parental activities but rather increase as a function of time spent at the nest, since food resources are limited near the nest. Leaving at all presumably greatly increases offspring mortality *via* predation (Segers, Gerber & Taborsky, 2011). As such, cost and benefit curves are likely to be closer to step functions (see Fig. S2). Body condition might be expected to decline linearly with time at the nest, but declining condition may in turn have a strongly accelerating negative effect on RRV. Manipulating food available at the nest site thus worked to alter desertion rates but not specific parental activities for two possibly fortuitous reasons. First, subjects were close to the threshold body condition for desertion (see Fig. S2) so that food supplements would actually have an effect. Second, subjects responded to cues of food near their nest by feeding, despite the fact that they were caught earlier using angling equipment (which mimics food). In other words, it is the mix of understanding the biology of cost and benefit functions and the types of cues used by subject decision‐making mechanisms that allow for successful experiments.

## V. CONCLUSIONS

(1) That parental care is a life‐history trait subject to trade‐offs has been the major organizing idea for explaining diversity of care. There is considerable circumstantial evidence supporting this idea, but the details have been challenging to test. Our review and meta‐analysis highlights both these circumstances.
(2) The main experimental approach in birds, brood size manipulations, offers support for the main hypothesis: when the value of current offspring changes, parents alter their care and they do so in a way that supports theory. However, other predictions from theory that might explain variation in parental responses were not supported, and the reasons for the lack of support remain unclear.
(3) We suggest that limitations of brood manipulations themselves or what we know about the context in which they were done may be part of the explanation. To that end, we reviewed other types of experiments that have altered care levels, from which two main conclusions emerged. First, most manipulations are not designed to allow tests of finer elements of life‐history theory, in part because many were used to address other questions. Second, we conclude that opportunities to test theory more effectively exist.
(4) To address these shortcomings, protocols that target a single trait and alter one fitness component in systems where key cues for plasticity in care are known are needed. These can be brought to bear on poorly tested aspects of foundational theory and the new hypotheses arising from more recent theory that has incorporated more realistic interactions among variables and feedbacks in both ecological and evolutionary time. To this end, studies of single populations will continue to be necessary and informative but are unlikely to be diagnostic on their own. We also emphasize the insights that can be gained from multiple studies across a wider array of species having different ecologies. Such work can inform new meta‐analyses that can test these new ideas on a broader scale.
(5) We conclude that new tests of parental care as a life‐history trait are warranted but may be most effective when approached with well‐stocked toolboxes containing detailed natural history, an awareness of the impact of mechanism on functional responses, good statistical approaches, and a deep understanding of theory.

## AUTHOR CONTRIBUTIONS

R.A.M. and K.J.M. conceived of the study. R.A.M., S.S., and K.J.M. performed the literature review. R.A.M. performed data extractions with support from K.J.M. R.A.M. and K.J.M. analysed the data, with support from M.L. R.A.M., D.F.W. and K.J.M. co‐wrote the first draft. All co‐authors contributed to revisions.

## Supporting information


**Appendix S1.** Search string details.
**Appendix S2.** Life‐history data.
**Table S1.** PRISMA Ecology and Evolution reporting checklist (based on O'Dea *et al*., 2021).
**Table S2.** Full list of studies (both peer‐reviewed publications and theses) that were excluded after reading the full text.
**Table S3.** Overview of the information extracted from each article included in the meta‐analysis.
**Table S4.** Data sources for bird species life‐history data used as potential moderators of effects in meta‐regression models.
**Table S5.** Total heterogeneity (*I* ^2^, which can vary between 0 and 100) and heterogeneity at each hierarchical level (fitted as random effects, see text for details) for meta‐analytic models using different phylogenetic relationships and different data sets [Complete *versus* Reduced, i.e. excluding the study with outliers: Ardia (2007)].
**Table S6.** Proportion of variation explained by different moderators when re‐running analyses presented in the main text with the alternative phylogenetic tree (Tree of Life, accessed March 3rd 2026), and with the Complete data set *versus* the Reduced data set that excluded one study with potential outliers (Ardia, 2007) (see Table S8).
**Table S7.** Estimated effects sizes (*β*) and 95% confidence intervals (CI) for different moderators when re‐running analyses presented in the main text with the alternative phylogenetic tree (Tree of Life, accessed March 3rd 2026), and with the Complete data set *versus* the Reduced data set that excluded one study with potential outliers (Ardia, 2007) (see Table S8).
**Table S8.** The 10 highest sign‐adjusted Hedges' *g* values (G_flip) and associated variance (vi) from our data set in order from highest to lowest (1 = highest, 10 = lowest of the 10), with Study ID (i.e. unique study identifier) and Study.
**Fig. S1.** Data visualizations to assess whether important methodological moderators were confounded with treatment or life‐history variables.
**Fig. S2.** Graphical model of optimal parental care when benefit and cost curves are strongly non‐linear (nearly step functions), which leads to threshold effects on care decisions.

## Data Availability

All data and code required to reproduce the analyses presented in the main text are archived on the Open Science Framework digital repository and can be accessed at: https://doi.org/10.17605/OSF.IO/7KYF2 (McKinnon *et al*., 2026).
