# Supplementary material for: Trade‐offs in avian parental care: a review of theory and meta‐analysis of brood size manipulations
Source: Biol Rev Camb Philos Soc. 2026 May 12;101(5):2337–58. doi: 10.1002/brv.70181 (PMC13542356; doi:10.1002/brv.70181)
Supplement: Supplementary file 1 [file BRV-101-2337-s001.docx]

Supporting information for: Trade-offs in avian parental care: a review of theory and meta-analysis of brood size manipulations

Table of Contents

**[Appendix S1](#_Toc223445080)**[. Search string details	2](#_Toc223445080)

**[Appendix S2](#_Toc223445081)**[. Life-history data	3](#_Toc223445081)

**[Table S1.](#_Toc223445082)** [PRISMA Ecology and Evolution reporting checklist (based on O'Dea](#_Toc223445082) *[et al.](#_Toc223445082)*[, 2021).	4](#_Toc223445082)

**[Table S2.](#_Toc223445083)** [Full list of studies (both peer-reviewed publications and theses) that were excluded after reading the full text. .	10](#_Toc223445083)

**[Table S3.](#_Toc223445084)** [Overview of the information extracted from each article included in the meta-analysis..	13](#_Toc223445084)

**[Table S4.](#_Toc223445085)** [Data sources for bird species life-history data used as potential moderators of effects in meta-regression models.	16](#_Toc223445085)

**[Table S5.](#_Toc223445086)** [Total heterogeneity (](#_Toc223445086)*[I](#_Toc223445086)*^[2](#_Toc223445086)^[, which can vary between 0 and 100) and heterogeneity at each hierarchical level (fitted as random effects, see text for details) for meta-analytic models using different phylogenetic relationships and different data sets [Complete](#_Toc223445086) *[versus](#_Toc223445086)* [Reduced, i.e. excluding study with outliers: Ardia (2007)]..	18](#_Toc223445086)

**[Table S6.](#_Toc223445087)** [Proportion of variation explained by different moderators when re-running analyses presented in the main text with the alternative phylogenetic tree (Tree of Life, accessed March 3rd 2026), and with the Complete data set](#_Toc223445087) *[versus](#_Toc223445087)* [the Reduced data set that excluded one study with potential outliers (Ardia, 2007) (see Table S8).	19](#_Toc223445087)

**[Table S7.](#_Toc223445088)** [Estimated effects sizes (β) and 95% confidence intervals (CI) for different moderators when re-running analyses presented in the main text with the alternative phylogenetic tree (Tree of Life, accessed March 3rd 2026), and with the Complete data set](#_Toc223445088) *[versus](#_Toc223445088)* [the Reduced data set that excluded one study with potential outliers (Ardia, 2007) (see Table S8).	20](#_Toc223445088)

**[Table S8.](#_Toc223445089)** [The 10 highest sign-adjusted Hedges’](#_Toc223445089) *[g](#_Toc223445089)* [values (G_flip) and associated variance (vi) from our data set in order from highest to lowest (1 = highest, 10 = lowest of the 10), with Study ID (i.e., unique study identifier) and Study.	22](#_Toc223445089)

**[Fig. S1.](#_Toc223445090)** [Data visualizations to assess whether important methodological moderators were confounded with treatment or life-history variables.	23](#_Toc223445090)

**[Fig. S2.](#_Toc223445091)** [Graphical model of optimal parental care when benefit and cost curves are strongly non-linear (nearly step functions), which leads to threshold effects on care decisions..	25](#_Toc223445091)

**[Supplemental references](#_Toc223445092)**	[26](#_Toc223445092)

# Appendix S1. Search string details

Below is the final search string used in the meta-analysis presented in the main text for *Web of Science* Core Collections. The search string had two groups of terms, one to capture parental effort, and one to capture brood size manipulations. Terms within each group were joined by the Boolean operator ‘OR’ while categories were joined by ‘AND’ such that a study needed to have a term from both the first and second category (parental effort and brood size manipulation, respectively) within its title, abstract or key words in order to be returned by the database that was being searched. This search string was originally formed within ‘advanced search’ in *Web of Science* and can be copied and pasted directly to reproduce our search.

Note: The equivalent search strings were used in each of our database searches (i.e. also for *Scopus*, *Worldwidescience*, and *BASE*) with edits to reflect the database ‘wildcard’ characters. For example, in *Web of Science* the asterisk symbol (*) can be used to capture any group of characters, including no character, i.e. “provision*” captures provision, provisioning, provisioned, provisions etc; and the dollar sign ($) can be used to capture any single character, i.e. “feeding visit$” captures feeding visit and feeding visits.

Wildcard characters are used for precisely the same meaning across *Web of Science* and *Scopus*. In *Worldwidescience* and *BASE* the use of ‘$’ is replaced by ‘?’. Descriptions of the wildcard usage for each of our searched databases can be found (linked) at: [Web of Science](https://images.webofknowledge.com/images/help/WOS/hs_wildcards.html), [Scopus](https://library-guides.ucl.ac.uk/scopus/search-techniques#:~:text=By%20default%2C%20Scopus%20automatically%20includes,%2C%20use%20%7Bcurly%20brackets%7D.), [worldwidescience](https://worldwidescience.org/help.html) and [BASE](https://www.searchsmart.org/results/base?~()).

The full search string is as follows:

(TS=(provision* OR "parental effort" OR "parental investment" OR "visit rate" OR "prey delivery" OR "energy delivery" OR "parental provision*" OR "feeding visit$" OR "feeding rate" OR "feeding effort"))

AND

(TS=("brood size" OR "nestling number" OR "number of nestlings" OR "reduced brood" OR "enlarged brood" OR "clutch size" OR "brood reduction" OR "brood enlargement" OR "number of chicks" OR "chick number" OR "brood demand"))

# Appendix S2. Life-history data

We were interested in testing the moderating effects of several life-history variables on the strength of response to brood size manipulations in birds. These variables included:

- mean clutch size (i.e. mean number of eggs per single clutch in a given species);
- age at first reproduction (i.e. species mean age at first reproduction, in months);
- annual survival probability for adults (note, because sex-specific survival estimates for adults were seldom available, we used an overall adult survival estimates for the species);
- broodedness (i.e. whether the study species/population regularly produces a single or multiple broods per reproductive season);
- mean number of broods in a year for the species.

Wherever possible, these life-history parameter values were extracted from the source article. When that was not possible, we obtained species/population-specific data where possible from Birds of the World (Billerman *et al.*, 2022) (for North American Species) or the British Trust for Ornithology (BTO, 2023) (for Eurasian species). When species-specific values were not available, we used family-level estimates from Appendix 1 in Bennett & Owens (2002). Sources for all life-history parameters used for each species are listed Table S4.

Annual fecundity was considered as an additional life-history variable that might shape responses to brood size manipulations, and was calculated as species- (or population-)specific clutch size multiplied by species-specific broods per year.

# Table S1. PRISMA Ecology and Evolution reporting checklist (based on O'Dea *et al.*, 2021).

| **Checklist item** | **Sub-item number** | **Sub-item** | **Reported by authors?** | **Notes** |
| --- | --- | --- | --- | --- |
| Title and Abstract | 1.1 | Identify the review as a systematic review, meta-analysis, or both | Yes | Title, Abstract |
|  | 1.2 | Summarise the aims and scope of the review | Yes | Abstract |
|  | 1.3 | Describe the data set | Yes | Abstract |
|  | 1.4 | State the results of the primary outcome | Yes | Abstract |
|  | 1.5 | State conclusions | Yes | Abstract |
|  | 1.6 | State limitations | Yes | Abstract |
| Aims and questions | 2.1 | Provide a rationale for the review | Yes | Introduction |
|  | 2.2 | Reference any previous reviews or meta-analyses on the topic | Yes | Introduction, Discussion |
|  | 2.3 | State the aims and scope of the review (including its generality) | Yes | Introduction |
|  | 2.4 | State the primary questions the review addresses (e.g. which moderators were tested) | Yes | Abstract, Introduction,  Methods |
|  | 2.5 | Describe whether effect sizes were derived from experimental and/or observational comparisons | Yes | Methods |
| Review registration | 3.1 | Register review aims, hypotheses (if applicable), and methods in a time-stamped and publicly accessible archive and provide a link to the registration in the methods section of the manuscript. Ideally registration occurs before the search, but it can be done at any stage before data analysis. | No |  |
|  | 3.2 | Describe deviations from the registered aims and methods | NA | Study was not pre-registered |
|  | 3.3 | Justify deviations from the registered aims and methods | NA | Study was not pre-registered |
| Eligibility criteria | 4.1 | Report the specific criteria used for including or excluding studies when screening titles and/or abstracts, and full texts, according to the aims of the systematic review (e.g. study design, taxa, data availability) | Yes | Methods |
|  | 4.2 | Justify criteria, if necessary (i.e. not obvious from aims and scope) | Yes | Methods |
| Finding studies | 5.1 | Define the type of search (e.g. comprehensive search, representative sample) | Yes | Methods |
|  | 5.2 | State what sources of information were sought (e.g. published and unpublished studies, personal communications) | Yes | Methods, Supporting information |
|  | 5.3 | Include, for each database searched, the exact search strings used, with keyword combinations and Boolean operators | Yes | Methods, Supporting information |
|  | 5.4 | Provide enough information to repeat the equivalent search (if possible), including the timespan covered (start and end dates) | Yes | Methods, Results, Supporting information |
| Study selection | 6.1 | Describe how studies were selected for inclusion at each stage of the screening process (e.g. use of decision trees, screening software) | Yes | Methods |
|  | 6.2 | Report the number of people involved and how they contributed (e.g. independent parallel screening) | Yes | Methods |
| Data collection process | 7.1 | Describe where in the reports data were collected from (e.g. text or figures) | Yes | Methods |
|  | 7.2 | Describe how data were collected (e.g. software used to digitize figures, external data sources) | Yes | Methods |
|  | 7.3 | Describe moderator variables that were constructed from collected data (e.g. number of generations calculated from years and average generation time) | Yes | Methods |
|  | 7.4 | Report how missing or ambiguous information was dealt with during data collection (e.g. authors of original studies were contacted for missing descriptive statistics, and/or effect sizes were calculated from test statistics) | Yes | Missing species-specific life-history (LH) data were estimated based on data from closely related species, described in the Supporting information |
|  | 7.5 | Report who collected data | Yes | Methods |
|  | 7.6 | State the number of extractions that were checked for accuracy by co-authors | Yes | Methods |
| Data items | 8.1 | Describe the key data sought from each study | Yes | Methods, Supporting information |
|  | 8.2 | Describe items that do not appear in the main results, or which could not be extracted due to insufficient information | Yes | Methods |
|  | 8.3 | Describe main assumptions or simplifications that were made (e.g. categorising both ‘length’ and ‘mass’ as ‘morphology’) | Yes | Methods, Supporting information |
|  | 8.4 | Describe the type of replication unit (e.g. individuals, broods, study sites) | Yes | Methods |
| Assessment of individual study quality | 9.1 | Describe whether the quality of studies included in the systematic review or meta-analysis was assessed (e.g. blinded data collection, reporting quality, experimental *vs*. observational) | No | All studies included were experimental, quality assessment not done |
|  | 9.2 | Describe how information about study quality was incorporated into analyses (e.g. meta-regression and/or sensitivity analysis) | NA | Information on quality was not incorporated into analyses |
| Effect size measures | 10.1 | Describe effect size(s) used | Yes | Methods |
|  | 10.2 | Provide a reference to the equation of each calculated effect size (e.g. standardised mean difference, log response ratio) and (if applicable) its sampling variance | Yes | Methods |
|  | 10.3 | If no reference exists, derive the equations for each effect size and state the assumed sampling distribution(s) | NA | A reference for the effect size and its sampling variance was available, so no derivation was required |
| Missing data | 11.1 | Describe any steps taken to deal with missing data during analysis (e.g. imputation, complete case, subset analysis) | Yes | Missing species-specific LH data were estimated based on data from closely related species, described in the Supporting information |
|  | 11.2 | Justify the decisions made to deal with missing data | Yes | Missing species-specific LH data were estimated based on data from closely related species, described in the Supporting information |
| Meta-analytic model description | 12.1 | Describe the models used for synthesis of effect sizes | Yes | Methods |
|  | 12.2 | The most common approach in ecology & evolution will be a random-effects model, often with a hierarchical/multilevel structure. If other types of models are chosen (e.g. common/fixed effects model, unweighted model), provide justification for this choice | NA | Hierarchical random effect models were used |
| Software | 13.1 | Describe the statistical platform used for inference (e.g. *R*) | Yes | Methods |
|  | 13.2 | Describe the packages used to run models | Yes | Methods |
|  | 13.3 | Describe the functions used to run models | Yes | Methods |
|  | 13.4 | Describe any arguments that differed from the default settings | NA | Arguments used did not differ from default settings |
|  | 13.5 | Describe the version numbers of all software used | Yes | Full code with version numbers for packages provided in public repository (OSF) [here](https://doi.org/10.17605/OSF.IO/7KYF2). |
| Non-independence | 14.1 | Describe the types of non-independence encountered (e.g. phylogenetic, spatial, multiple measurements over time) | Yes | Methods |
|  | 14.2 | Describe how non-independence has been handled | Yes | Methods |
|  | 14.3 | Justify decisions made | Yes | Methods |
| Meta-regression and model selection | 15.1 | Provide a rationale for the inclusion of moderators (covariates) that were evaluated in meta-regression models | Yes | Methods |
|  | 15.2 | Justify the number of parameters estimated in models, in relation to the number of effect sizes and studies (e.g. interaction terms were not included due to insufficient sample sizes) | No | Justification not provided. Number of parameters estimated was small relative to sample size. |
|  | 15.3 | Describe any process of model selection | Yes | Methods |
| Publication bias and sensitivity analyses | 16.1 | Describe assessments of the risk of bias due to missing results (e.g. publication, time-lag, and taxonomic biases) | Yes | Methods |
|  | 16.2 | Describe any steps taken to investigate the effects of such biases (if present) | Yes | Methods |
|  | 16.3 | Describe any other analyses of robustness of the results, e.g. due to effect size choice, weighting or analytical model assumptions, inclusion or exclusion of subsets of the data, or the inclusion of alternative moderator variables in meta-regressions | Yes | Methods/Supporting information |
| Clarification of *post hoc* analyses | 17.1 | When hypotheses were formulated after data analysis, this should be acknowledged | NA | No hypotheses formulated after data analysis |
| Metadata, data, code | 18.1 | Share metadata (i.e. data descriptions) | Yes | Supporting information |
|  | 18.2 | Share data required to reproduce the results presented in the manuscript | Yes | All data/code is archived on OSF [here](https://doi.org/10.17605/OSF.IO/7KYF2). |
|  | 18.3 | Share additional data, including information that was not presented in the manuscript (e.g. raw data used to calculate effect sizes, descriptions of where data were located in papers) | Yes | All data/code is archived on OSF [here](https://doi.org/10.17605/OSF.IO/7KYF2). |
|  | 18.4 | Share analysis scripts (or, if a software package with graphical user interface (GUI) was used, then describe full model specification and fully specify choices) | Yes | All data/code is archived on OSF [here](https://doi.org/10.17605/OSF.IO/7KYF2). |
| Results of study selection process | 19.1 | Report the number of studies screened | Yes | Results |
|  | 19.2 | Report the number of studies excluded at each stage of screening | Yes | Results, Supporting information |
|  | 19.3 | Report brief reasons for exclusion from the full-text stage | Yes | Supporting information |
|  | 19.4 | Present a Preferred Reporting Items for Systematic Reviews and Meta-Analyses (PRISMA)-like flowchart ([www.prisma-statement.org](http://www.prisma-statement.org)) | Yes | Results |
| Sample sizes and study characteristics | 21.1 | Provide a summary of key characteristics for reported outcomes (either in text or figures; e.g. one quarter of effect sizes reported for vertebrates and the rest invertebrates) | Yes | Results, Figures |
|  | 20.2 | Report the number of studies and effect sizes for subsets of data included in meta-regressions | Yes | Figures |
|  | 20.3 | Provide a summary of key characteristics for reported outcomes (either in text or figures; e.g. one quarter of effect sizes reported for vertebrates and the rest invertebrates) | Yes | Results |
|  | 20.4 | Provide a summary of limitations of included moderators (e.g. collinearity and overlap between moderators) | Yes | Results, Supporting information, Discussion |
|  | 20.5 | Provide a summary of characteristics related to individual study quality (risk of bias) | NA | The quality of studies was not included in the meta-analysis |
| Meta-analysis | 21.1 | Provide a quantitative synthesis of results across studies, including estimates for the mean effect size, with confidence/credible intervals | Yes | Results |
| Heterogeneity | 22.1 | Report indicators of heterogeneity in the estimated effect (e.g. *I*^2^, tau^2^ and other variance components) | Yes | Results, Supporting information |
| Meta-regression | 23.1 | Provide estimates of meta-regression slopes (i.e. regression coefficients) and confidence/credible intervals | Yes | Results, Supporting information |
|  | 23.2 | Include estimates and confidence/credible intervals for all moderator variables that were assessed (i.e. complete reporting) | Yes | Results, Supporting information |
|  | 23.3 | Report interactions, if they were included | Yes | Methods, Results, Supporting information |
|  | 23.4 | Describe outcomes from model selection, if done (e.g. *R*^2^ and AIC) | NA | Model selection not performed, all model results reported |
| Outcomes of publication bias & sensitivity analyses | 24.1 | Provide results for the assessments of the risks of bias (e.g. Egger’s regression, funnel plots) | Yes | Results, Supporting information |
|  | 24.2 | Provide results for the robustness of the review’s results (e.g. subgroup analyses, meta-regression of study quality, results from alternative methods of analysis, and temporal trends) | Yes | Results, Supporting information, Discussion |
| Discussion | 25.1 | Summarise the main findings in terms of the magnitude of effect | Yes | Discussion |
|  | 25.2 | Summarise the main findings in terms of the precision of effects (e.g. size of confidence Summarise the main findings in terms of the precision of effects (e.g. size of confidence intervals, statistical significance) | Yes | Discussion |
|  | 25.3 | Summarise the main findings in terms of their heterogeneity | Yes | Discussion |
|  | 25.4 | Summarise the main findings in terms of their biological/practical relevance | Yes | Discussion |
|  | 25.5 | Compare results with previous reviews on the topic, if available | Yes | Discussion |
|  | 25.6 | Consider limitations and their influence on the generality of conclusions, such as gaps in the available evidence (e.g. taxonomic and geographical research biases) | yes | Discussion |
| Contributions and funding | 26.1 | Provide names, affiliations, and funding sources of all co-authors | Yes |  |
|  | 26.2 | List the contributions of each co-author | Yes | Methods, Author contributions |
|  | 26.3 | Provide contact details for the corresponding author | Yes |  |
|  | 26.4 | Disclose any conflicts of interest | Yes | There were no conflicts of interest |
| References | 27.1 | Provide a reference list of all studies included in the systematic review or meta-analysis | Yes | Marked with * in reference list in main paper |

# Table S2. Full list of studies (both peer-reviewed publications and theses) that were excluded after reading the full text. Search criteria (A, B, C, or D) corresponds to the search labels in the PRISMA flow chart (Fig. 2 in main paper). Full references of all studies are provided at the end of this document. BSM, brood size manipulation.

| **Reference** | **Search** | **Reason** |
| --- | --- | --- |
| Aho (1997) | C | Publication from thesis captured in search A |
| Allen *et al.* (2023) | B | No control |
| Arct *et al.* (2022) | D | No provisioning behaviour |
| Arnold (1993) | A | No provisioning data |
| Arnold (2011) | A | No provisioning data |
| Beissinger (1990) | A | Provisioning data not linked to BSM |
| Boonekamp, Bauch & Verhulst (2020) | D | No provisioning behaviour |
| Boonekamp *et al.* (2014) | D | No provisioning behaviour |
| Bowers *et al.* (2014) | A | No control |
| Cauchard *et al.* (2017) | A | No BSM |
| Conrad & Robertson (1993) | A | Provisioning data presented only at per nestling level |
| Cornelius Ruhs *et al.* (2020) | D | Insufficient sample sizes (3 control nests, 2 reduced nests, 2 enlarged nests) |
| Costanzo *et al.* (2016) | D | No provisioning behaviour |
| Cucco & Malacarne (1995) | A | Insufficient sample size (*N* = 2 control) |
| Dawson (1999) | C | Publication from thesis captured in search A |
| Dykstra & Karasov (1993) | A | Provisioning data not linked to BSM |
| Edge, Jamieson & Darby (1999) | A | No provisioning data |
| Emaresi *et al.* (2014) | A | Provisioning data not linked to BSM |
| Engelbretsen & Slagsvold (2023) | B | No control |
| Ferretti, Llambias & Martin (2005) | A | No provisioning data |
| Fokkema, Ubels & Tinbergen (2016) | A | Provisioning data not linked to BSM |
| Galbraith (1983) | A | No provisioning data |
| Garcia-Campa *et al.* (2023) | B | No control |
| Gebczynski, Taylor & Konarzewski (1996) | A | No provisioning data |
| González-Medina, Castillo-Guerrero & Mellink (2010) | A | Natural and experimental broods pooled |
| Gori (1988) | A | Manipulations applied non-randomly |
| Gow (2014) | C | Publication from thesis captured in search A |
| Gow, Wiebe & Musgrove (2015) | A | No provisioning data |
| Green & Ydenberg (1994) | A | Extractions not possible |
| Gustafsson, Qvarnstrom & Sheldon (1995) | A | No provisioning data |
| Hainstock *et al.* (2010) | A | Extractions not possible |
| Heaney & Monaghan (1995) | A | No control |
| Heaney (1997) | C | No control (Chp. 3), No provisioning (Chp. 4, 5, 6), Multiple manipulations (Chp 7) |
| Hegner & Wingfield (1987) | A | Extractions not possible |
| Hinde & Kilner (2006) | A | Extractions not possible (data presented based on final brood size, not manipulation) |
| Ibáñez-Álamo, Arco & Soler (2012) | A | No control |
| Jelinek *et al.* (2016) | A | No BSM |
| Jones (1987) | A | Extractions not possible (data presented based on final brood size, not manipulation) |
| Klemp (2000) | A | No control |
| Leach *et al.* (2019) | D | Non-random BSM |
| Leffelaar & Robertson (1986) | A | Extractions not possible (no variances provided) |
| Leonard *et al.* (2000) | A | Additional manipulations conducted |
| Lifjeld & Slagsvold (1991) | A | Additional manipulations conducted |
| Lozano & Lemon (1998) | A | No control |
| Lucass *et al.* (2016) | A | No BSM |
| Mariette & Griffith (2015) | A | No control |
| Martins & Wright (1993*a*) | A | Extractions not possible (data presented based on final brood size, not manipulation) |
| Martins & Wright (1993*b*) | A | Extractions not possible (data presented based on final brood size, not manipulation) |
| Martins (1997) | A | No provisioning data |
| Moreno (1987) | A | Extractions not possible (data presented based on final brood size, not manipulation) |
| Murphy *et al.* (2000) | A | Extractions not possible (data presented based on final brood size, not manipulation) |
| Musgrove (2014) | C | Publication from thesis captured in search A |
| Nur (1984) | A | Extractions not possible (data presented based on final brood size, not manipulation) |
| Olsen & Tucker (2003) | A | Sample sizes insufficient (*N* = 2 enlarged, *N* = 2 control, *N* = 1 reduced) |
| Ostreiher & Heifetz (2015) | A | BSM non-random |
| Patterson, Erckmann & Orians (1980) | A | Inappropriate design (males had multiple nests, so looking at effect of brood reduction at one nest on investment at others) |
| Pryke & Griffith (2009) | A | No control |
| Przybylo & Merilä (2000) | A | No control |
| Ridenour (2020) | C | No control |
| Roulin, Ducrest & Dijkstra (1999) | A | No control |
| Rytkonen (1995) | A | Extractions not possible |
| Saino *et al.* (2000) | A | No control |
| Saino *et al.* (2018) | D | No provisioning behaviour |
| Saino, Calza & Moller (1997) | A | No control |
| Sendecka, Cichon & Gustafsson (2007) | A | No provisioning data |
| Shutler *et al.* (2006) | A | Extractions not possible (data presented based on final brood size, not manipulation) |
| Siefferman & Hill (2005) | A | No control |
| Siefferman & Hill (2007) | A | No control |
| Siikamaki *et al.* (1998) | A | No appropriate response variable |
| Simmons (1986) | A | No BSM |
| Slagsvold & Rohwer (2000) | A | No control |
| Sousa (2008) | C | Publication from thesis captured in search A |
| Styrsky, Brawn & Robinson (2005) | A | Provisioning data presented only at per nestling level |
| Sudyka *et al.* (2016) | D | No provisioning behaviour |
| Thomson *et al.* (2014) | D | No provisioning behaviour |
| Ursino (2016) | C | Publication from thesis captured in search A |
| Westneat *et al.* (2017) | A | No control |
| Whittingham (1989) | A | Provisioning data not linked to BSM |
| Wiersma & Tinbergen (2003) | A | No control |
| Wright & Cuthill (1990) | A | Extractions not possible (data presented based on final brood size, not manipulation) |
| Wright *et al.* (1998) | A | Extractions not possible (data presented based on final brood size, not manipulation) |
| Wright *et al.* (2003) | A | No control |

# Table S3. Overview of the information extracted from each article included in the meta-analysis. IVI, inter-visit interval. See files deposited on OSF at the following link for the full datasheet used: <https://doi.org/10.17605/OSF.IO/7KYF2> (McKinnon *et al.*, 2026).

| **Column heading** | **Details** |
| --- | --- |
| StudyID | Represents the unique record number of that study in the EndNote library used for management of all studies included in this meta-analysis. |
| Author | Family name of author(s) for that study. We recorded the family names for up to 2 authors (e.g. Berzins&Dawson), but if the study had more than 2 authors, we recorded the family name of the first author followed by et al. (e.g. Baldan et al). |
| Year | Year of study publication. Recorded as YYYY format. |
| FocalSpC | Common name of the species investigated in that study. Recorded in sentence case and as plurals, e.g. Eastern phoebes not Eastern Pheobe. |
| FocalSpL | Latin name of the species investigated in that study. Recorded in sentence case, e.g. Sayornis phoebe. |
| Treatment | Treatment type considered in the given treatment–control comparison. We used two levels: experimental brood enlargement (‘enlarged’) or experimental brood reduction (‘reduced’). When a study had two types of treatment, they were extracted separately and expressed as separate effect sizes (treatment–control comparisons). |
| Treatment(details) | Number of eggs or nestlings added or removed as a short sentence/phrase. For example, ‘remove *x* eggs’ or ‘add 2 nestlings’ according to what was conducted in the study. This information is also reflected in other columns i.e. Treatment (for whether eggs/nestlings were added or removed), and Treatment_stage (for egg or nestling, see below). |
| Fixed_Variable_Manip | Whether the experimental manipulation conducted involved a fixed number of additions/removals at each nest, or a variable number of additions/removals depending on the natural characteristics of each nest. For example, many studies removed 2 nestlings from all nests and recorded the brood size created, but some removed either 1 or 2 nestlings to create broods of a specific size. In the latter case, even though the magnitude of manipulation was non-random, the selection of nests and assignment to enlargement or reduction treatments was random. |
| Treatment_CONT | The number of eggs/nestlings added/removed in the treatment group per nest as a numerical value. If a variable number of eggs/nestlings were added/removed, the average value was recorded. For example, if Treatment(details) recorded ‘remove 2–3 nestlings’ then 2.5 was recorded in this column. |
| Control(details) | Recorded as TRUE (for a true control) or FALSE. A true control was one in which the control nests were handled in the same manner and at the same regularity as the treatment nests. For example, nestlings or eggs may have been swapped for control nests while keeping the brood/clutch size unchanged. FALSE indicates that unmanipulated nests served as controls. |
|  |  |
| ClutchSize | Mean clutch size for the species/population. Data extracted from the included study wherever possible, otherwise extracted from other sources as described in Appendix S2. |
| Control_BSorCS | Average control brood (or clutch) size recorded as a numerical value. When manipulations were conducted at the egg stage, the value is a clutch size; when manipulations were conducted at the nestling stage, the value is brood size. This allowed us to calculate the relative magnitude of the manipulations. This information was recorded from the study when available, otherwise, it was extracted from Cornell All About Birds (Cornell Lab of Ornithology, 2025). |
| SourceOfBS | Source of control_broodsize information, i.e. ‘study’ if this information was reported in the study itself, ‘prev study’ if this information was recorded based on what was presented in a different study on the same species, or ‘cornell’ if this information was recorded from Cornell All About Birds (Cornell Lab of Ornithology, 2025). |
| Relchange | Relative change in brood size for the manipulations expressed as the proportion of natural brood size. Calculated as number of egg/nestlings added (enlarged broods) or removed (reduced broods) divided by the mean control brood size. |
| Treatment_stage | Stage at which the brood size manipulation was conducted, i.e. ‘egg’ if eggs were added/removed, or ‘nestling’ if nestlings were added/removed. |
| Treat_dur_min | Minimum number of days between the start of the brood size manipulation and behavioural observations. Expressed in units of days. When the observations commenced on the same day as the treatment was carried out, Treat_dur_min is 0. |
| TreatDurCat | Treatment duration categorized into either ‘long’ term or ‘short’ term. When behavioural observations occurred only on the same day as the manipulation, the study duration category was recorded as ‘short’. Where observations were made repeatedly across the breeding period over multiple days it was recorded as ‘long’. * |
| RespCat | Response variable investigated in that study categorised as either feeding rate (FeedingRate), visit rate (VisitRate), or biomass delivery, a measure of size or mass delivered per unit time (Delivery) |
| ResponseVar | Details of the measured provisioning response of the parents, within each response category, i.e. feeds/visits per unit time, IVI, prey size, load size. |
| StudyDesign | Whether the study (i.e. brood manipulation) was conducted within or among individuals/subjects. Some studies conducted within-subject manipulations, for example measuring the feeding rate to the nest before adding/removing nestlings (as the control) and then again after adding/removing nestlings at the same nest; these were recorded as ‘within.’ Other (most) studies conducted among-subject manipulations with some nests acting as control nests and some as treatment nests throughout the study period (recorded as ‘among’). |
| Nind_control | Number of provisioning parents total across all control nests, i.e. for biparental care species, 2 per nest; for uniparental care, 1 per nest. For species with uniparental care or in species where sex of provisioning parent cannot be teased apart (e.g. due to division of labour like in Peregrine falcons) Nind_control is equal to Nnests_control (below). |
| Nnests_control | Total number of control nests (i.e. nests without enlargement or reduction of the brood size used as comparator for the effects of brood size manipulation). |
| Nind_treat | Number of provisioning parents across all nests for that brood manipulation group (see ‘Treatment’ above), i.e. for biparental care species, 2 per nest; for uniparental care, 1 per nest. For species with uniparental care or in species where sex of provisioning parent cannot be teased apart Nind_treat is equal to Nnests_treat (below). |
| Nnests_treat | Total number of nests to which brood manipulation (enlargement or reduction) was applied, i.e. for rows recorded as ‘enlarged’ in the Treatment column (see above) this is the number of nests at which brood enlargement occurred. |
| Sex | Sex of the focal provisioning parent, i.e., male, female or both. We used ‘both’ if the study only reported total feeding rate irrespective of parental sex. |
| ES_flip | To aid interpretation of the results, positive effect sizes should reflect a change in the direction predicted by theory. We thus coded effect sizes which needed to be ‘flipped’ (to make their effect positive if aligning with theory) as a – 1, and those which did not need to be flipped as a 1. For example, IVIs are predicted to decrease at the level of the nest (reflecting an increase in provisioning rate) as brood demand increases (*via* brood enlargement) so we had to flip rows meeting this criterion so that a decrease in IVI at an enlarged brood would be presented as a positive effect size. To flip the sign of the relevant effect sizes we multiplied them by the value in this column (ES_flip). |
| Control_Mean | Mean outcome measure estimate for control group. |
| Control_SD | Standard deviation (SD) and standard error (SE), respectively, for the control group mean outcome measure. When it was only possible to extract one of these from the study (e.g. from a figure using WebPlotDigitizer, or from results text), we calculated the other using the equation SE = SD/√*N*, where *N* = number of nests (i.e. Nnests_control, see above). |
| Control_SE |  |
| Treatment_Mean | Mean outcome measure estimate for the treatment group. |
| Treatment_SD | Standard deviation (SD) and standard error (SE), respectively, for the relevant treatment group mean outcome mesure. When it was only possible to extract one of these from the study (e.g. from a figure using WebPlotDigitizer, or from results text), we calculated the other using the equation SE = SD/√*N*, where *N* = number of nests (i.e. Nnests_treat, see above). |
| Treatment_SE |  |
| Notes | Any additional notes relevant to data extraction, e.g., which figures (and/or parts of figures) data was extracted from and exactly what data were extracted from that figure (i.e. mean and SE or mean and SD, etc.). |

*It should be noted that all ‘short’ term studies were also those conducted at the within-individual level (see ‘StudyDesign’) with complete overlap between the two (i.e. all within-individual studies were short-term and all short-term studies were at the within-individual level). Thus, ‘TreatDurCat’ is equivalent to ‘StudyDesign’ in this review.

# Table S4. Data sources for bird species life-history data used as potential moderators of effects in meta-regression models. AS = adult survival; CS = clutch size; AFR = Age at first reproduction; BPY = Broods per year; MA = maximum age in years (USGS = US Geological Survey bird database by Bird Banding Laboratory; EURING = Central Data Bank of the European Union for Bird Ringing; AnAge = The Animal Ageing and Longevity Database).

| **Common name** | **Latin Name** | **References** |
| --- | --- | --- |
| American kestrel | *Falco sparverius* | AS: (Bennett & Owens, 2002)  CS, AFR, BPY: (Smallwood & Bird, 2020)  MA: USGS |
| Barn swallow | *Hirundo rustica* | AS, CS, AFR, BPY: (Brown & Brown, 2020)  MA: EURING |
| Black guillemot | *Cepphus grylle* | AS, CS, AFR, BPY: (Butler *et al.*, 2020)  MA: USGS |
| Blue tit | *Parus caeruleus*/ *Cyanistes caeruleus* | AS, CS, AFR: (BTO, 2023)  BPY: (Bennett & Owens, 2002)  MA: EURING |
| Brown pelican | *Pelecanus occidentalis* | AS: (Bennett & Owens, 2002)  CS, AFR, BPY: (Shields, 2020)  MA: USGS |
| Cattle egret | *Bubulcus ibis* | AS: (Bennett & Owens, 2002)  CS, AFR, BPY: (Raymond, 2020)  MA: USGS |
| Collared flycatcher | *Ficedula albicollis* | AS, AFR, BPY: (Bennett & Owens, 2002)  CS: (Gustafsson & Nilsson, 1985)  MA: EURING |
| Eastern kingbird | *Tyrannus tyrannus* | AS, AFR, BPY: (Bennett & Owens, 2002)  CS: (Murphy & Pyle, 2020)  MA: USGS |
| Eastern phoebe | *Sayornis phoebe* | AS, CS, AFR, BPY: (Weeks Jr., 2020)  MA: USGS |
| Eurasian kestrel | *Falco tinnunculus* | AS, CS, AFR, BPY: (BTO, 2023)  MA: EURING |
| European starling | *Sturnus vulgaris* | AS, CS, AFR: (BTO, 2023)  BPY: (Bennett & Owens, 2002)  MA: USGS |
| Fairy martin | *Hirundo ariel* | CS: (Turner, 2020)  AS, AFR, BPY: (Bennett & Owens, 2002)  MA: AnAge |
| Great skua | *Catharacta skua* | CS, AS, AFR, BBY: (Furness, Boesman & Garcia, 2020)  MA: EURING |
| Great tit | *Parus major* | AS, CS, AFR: (BTO, 2023)  BPY: (Bennett & Owens, 2002)  MA: EURING |
| House sparrow | *Passer domesticus* | AFR, BPY (Bennett & Owens, 2002)  CS, AS: (Lowther & Cink, 2020)  MA: USGS |
| Lesser elaenia | *Elaenia chiriquensis* | CS: (Sousa & Marini, 2013)  AS, AFR, BPY: (Bennett & Owens, 2002)  MA: AnAge |
| Marsh tit | *Parus palustris* | AS, CS, AFR: (BTO, 2023)  BPY: (Bennett & Owens, 2002)  MA: EURING |
| Northern flicker | *Colaptes auratus* | AS, CS, AFR, BPY: (Wiebe & Moore, 2020)  MA: USGS |
| Pallid swift | *Apus pallidus* | CS: (Chantler, Bonan & Boesman, 2020)  AS, AFR, BPY: (Bennett & Owens, 2002)  MA: EURING |
| Pied flycatcher | *Ficedula hypoleuca* | CS. AS, AFR: (BTO, 2023)  BPY: (Bennett & Owens, 2002)  MA: EURING |
| Red-flanked bluetail | *Tarsiger cyanurus* | CS: (Collar *et al.*, 2020)  AS, AFR, BPY: (Bennett & Owens, 2002)  MA: EURING |
| Red-tailed tropicbird | *Phaethon rubricauda* | AS, AFR, CS, BPY: (Schreiber & Schreiber, 2020)  MA: AnAge |
| Red-winged blackbird | *Agelaius phoeniceus* | AS, CS, AFR, BPY: (Yasukawa & Searcy, 2020)  MA: USGS |
| Ring-billed gull | *Larus delawarensis* | AS: (Bennett & Owens, 2002)  CS, AFR, BPY: (Pollet *et al.*, 2020)  MA: USGS |
| Savannah sparrow | *Passerculus sandwichensis* | AS, CS, AFR, BPY: (Wheelwright & Rising, 2020)  MA: USGS |
| Southern swallow | *Tachycineta leucorrhoa* | AFR: (Bulit & Massoni, 2011)  AS: (Bennett & Owens, 2002)  CS, BPY: (Massoni & Reboreda, 2006)  MA: AnAge |
| Spotless starling | *Sturnus unicolor* | AS, CS, AFR, BPY: (BTO, 2023) records for *Sturnus vulgaris*  MA: EURING |
| Tawny owl | *Strix aluco* | AS, CS, AFR, BPY: (BTO, 2023)  MA: EURING |
| Tree swallow | *Tachycineta bicolor* | AS, CS, AFR, BPY: (Winkler *et al.*, 2020)  MA: USGS |
| Treecreeper | *Certhia familiaris* | AS, CS, AFR, BPY: (BTO, 2023)  MA: EURING |
| Willow tit | *Parus montanus* | AS, CS, AFR, BPY: (BTO, 2023)  MA: EURING |

# Table S5. Total heterogeneity (*I*^2^, which can vary between 0 and 100) and heterogeneity at each hierarchical level (fitted as random effects, see text for details) for meta-analytic models using different phylogenetic relationships and different data sets [Complete *versus* Reduced, i.e. excluding study with outliers: Ardia (2007)]. Note, we used two separate null meta-analytic models to ensure interpretations with respect to the effect of phylogeny were robust to different phylogenetic trees given uncertainty in some branches for avian phylogenies. The Tree of Life Phylogeny was accessed on 3rd March 2026. We also compared results with *versus* without estimates derived from Ardia (2007), to ensure inferences were not influenced by outliers (see Table S7).

| **Data set** | **Phylogeny** | ***I*^2^_[StudyID]_** | ***I*^2^_[estimateID]_** | ***I*^2^_[species]_** | ***I*^2^_[phylogeny]_** | ***I*^2^_[Total]_** |
| --- | --- | --- | --- | --- | --- | --- |
| **Complete** | **Tree of Life phylogeny** | 41.15 | 5.15 | <0.001 | 1.57 | 47.87 |
| **Complete** | **Jetz phylogeny** | 38.15 | 5.13 | <0.001 | 5.18 | 48.45 |
| **Reduced** | **Tree of Life phylogeny** | 23.87 | 3.18 | <0.001 | 7.90 | 34.96 |
| **Reduced** | **Jetz phylogeny** | 21.93 | 3.56 | <0.001 | 8.75 | 34.24 |

# Table S6. Proportion of variation explained by different moderators when re-running analyses presented in the main text with the alternative phylogenetic tree (Tree of Life, accessed March 3rd 2026), and with the Complete data set *versus* the Reduced data set that excluded one study with potential outliers (Ardia, 2007) (see Table S8).

| **Moderator** | **Results in main text:**  **Jetz phylogeny,**  **Complete data set** | **Tree Of Life phylogeny,**  **Complete data set** | **Jetz phylogeny,**  **Reduced data set** | **Tree Of Life phylogeny,**  **Reduced data set** |
| --- | --- | --- | --- | --- |
| ***Methodological*** | **Marginal *R*^2^** | **Marginal *R*^2^** | **Marginal *R*^2^** | **Marginal *R*^2^** |
| Relative change | 18.10 | 16.59 | 27.15 | 23.22 |
| Treatment * Relative change | 20.89 | 19.36 | 31.83 | 27.13 |
| Treatment stage (egg *versus* nestling) | 1.66 | 1.97 | 5.26 | 5.88 |
| Treatment duration (long *versus* short) | 0.19 | 0.14 | 1.17 | 1.11 |
| Response variable category (visit rate, feeding rate, or delivery) | 4.74 | 4.74 | 5.97 | 6.17 |
| Study design (within or among-subject) | 0.24 | 0.19 | 0.92 | 0.94 |
| Sex (male, female, or both combined) | 0.17 | 0.20 | 0.11 | 0.13 |
| **Life history** |  |  |  |  |
| Treatment | 1.97 | 1.97 | 4.08 | 3.88 |
| Treatment*Adult survival probability | 2.42 | 1.99 | 4.96 | 4.17 |
| Treatment*Clutch size | 2.19 | 2.42 | 4.75 | 6.54 |
| Treatment*Broods per year | 3.54 | 3.44 | 7.40 | 8.44 |
| Treatment*Lifetime productivity | 4.81 | 4.84 | 12.55 | 13.41 |

# Table S7. Estimated effects sizes (β) and 95% confidence intervals (CI) for different moderators when re-running analyses presented in the main text with the alternative phylogenetic tree (Tree of Life, accessed March 3rd 2026), and with the Complete data set *versus* the Reduced data set that excluded one study with potential outliers (Ardia, 2007) (see Table S8).

|  |  | **Results in main text:**  **Jetz phylogeny,**  **Complete data set** | **Tree Of Life Phylogeny,**  **Complete data set** | **Jetz phylogeny,**  **Reduced data set** | **Tree Of Life Phylogeny,**  **Reduced data set** |
| --- | --- | --- | --- | --- | --- |
| ***Methodological*** | **Model estimate** | **β (95% CI)** | **β (95% CI)** | **β (95% CI)** | **β (95% CI)** |
| Relative change  *Intercept estimated for treatment = Enlarged* | Intercept  Relative change | 0.37 (0.16, 0.57)  0.18 (0.09, 0.27) | 0.38 (0.11, 0.65)  0.18 (0.08, 0.27) | 0.33 (0.14, 0.51)  0.17 (0.09, 0.25) | 0.34 (0.08, 0.60)  0.17 (0.09, 0.24) |
| Treatment (E = enlarged, R = reduced) * Relative change  *Intercept estimated for treatment = E, and relative change = 0*  *Separate slopes estimated for the effect of manipulation magnitude (Rel_change) for each treatment level* | Intercept  Treatment(R)  Rel_change : E  Rel_change : R | 0.34 (0.14, 0.53)  0.13 (0.03, 0.23)  0.13 (0.03, 0.23)  0.24 (0.13, 0.35) | 0.35 (0.10, 0.60)  0.13 (0.03, 0.23)  0.13 (0.02, 0.23)  0.24 (0.13, 0.35) | 0.28 (0.10, 0.47)  0.14 (0.04, 0.24)  0.13 (0.04, 0.22)  0.23 (0.13, 0.33) | 0.30 (0.04, 0.56)  0.14 (0.04, 0.24)  0.12 (0.03, 0.21)  0.23 (0.13, 0.32) |
| Treatment stage (egg *versus* nestling)  *Intercept estimated for Stage = Egg* | Intercept  Stage (nestling) | 0.53 (0.20, 0.86)  –0.14 (–0.45, 0.17) | 0.56 (0.21, 0.92)  –0.16 (–0.47, 0.015) | 0.53 (0.26, 0.79)  –0.19 (–0.42, 0.05) | 0.57 (0.26, 0.87)  –0.21 (–0.45, 0.03) |
| Treatment duration (long *versus* short)  *Intercept estimated for Duration = Long* | Intercept  Duration(short) | 0.41 (0.22, 0.59)  0.04 (–0.22, 0.29) | 0.44 (0.27, 0.62)  0.03 (–0.23, 0.29) | 0.35 (0.16, 0.53)  0.07 (–0.13, 0.27) | 0.37 (0.15, 0.60)  0.07 (–0.13, 0.27) |
| Response variable category (visit rate (VR), feeding rate (FR), or delivery (Intercept))  *Intercept estimated for Response = Delivery* | Intercept  ResponseVar(FR)  ResponseVar(VR) | 0.13 (–0.18, 0.44)  0.33 (0.06, 0.59)  0.25 (–0.08, 0.59) | 0.14 (–0.18, 0.47)  0.33 (0.07, 0.59)  0.26 (–0.07, 0.60) | 0.12 (–0.16, 0.40)  0.27 (0.02, 0.52)  0.27 (–0.04, 0.56) | 0.14 (–0.17, 0.44)  0.28 (0.03, 0.53)  0.27 (–0.03, 0.58) |
| Study design (within- or among-subject)  *Intercept estimated for Study design = among-subject* | Intercept  StudyDesign(Within) | 0.41 (0.23, 0.59)  0.04 (–0.23, 0.31) | 0.45 (0.29, 0.60)  0.04 (–0.23, 0.31) | 0.35 (0.18, 0.53)  0.06 (–0.14, 0.27) | 0.38 (0.17, 0.59)  0.07 (–0.14, 0.27) |
| Sex (male, female, or both combined)  *Intercept estimated for sex = both combined* | Intercept  Sex(Female)  Sex(Male) | 0.42 (0.23, 0.60)  –0.01 (–0.17, 0.16)  0.03 (–0.14, 0.20) | 0.45 (0.28, 0.61)  0.00 (–0.17, 0.16)  0.04 (–0.13, 0.20) | 0.36 (0.19, 0.54)  –0.01 (–0.15, 0.14)  0.22 (–0.14, 0.17) | 0.39 (0.18, 0.59)  0.00 (–0.15, 0.15)  0.02 (–0.13, 0.18) |
| **Life history** |  |  |  |  |  |
| Treatment (E= enlarged, R = reduced)  *Intercept estimated for treatment = E* | Intercept  Treatment(R) | 0.36 (0.18, 0.54)  0.11 (0.01, 0.21) | 0.39 (0.19, 0.58)  0.11 (0.01, 0.21) | 0.30 (0.13, 0.48)  0.12 (0.02, 0.22) | 0.33 (0.12, 0.55)  0.12 (0.02, 0.22) |
| Treatment (E = enlarged, R = reduced)  * Adult survival probability (AS) (centred and standardized)  *Intercept estimated for Treatment = E, and for mean adult survival probability, Separate slopes estimated for the effect of Adult survival for each Treatment level* | Intercept  Treatment(R)  E : AS  R : AS | 0.32 (0.09, 0.55)  0.10 (0.00, 0.20)  0.05 (–0.07, 0.18)  0.00 (–0.14, 0.14) | 0.38 (0.14,0.61)  0.10 (0.00, 0.20)  0.03 (–0.10, 0.15)  –0.03 (–0.17, 0.10) | 0.28 (0.08, 0.48)  0.11 (0.01, 0.21)  0.06 (–0.05, 0.17)  –0.01 (–0.13, 0.11) | 0.33 (0.10, 0.56)  0.11 (0.01, 0.21)  0.03 (–0.08, 0.14)  –0.04 (–0.16, 0.08) |
| Treatment (E = enlarged, R = reduced)  *Clutch size (centred and standardized)  *Intercept estimated for Treatment = E, and for mean clutch size, Separate slopes estimated for the effect of Clutch size for each Treatment level* | Intercept  Treatment(R)  E : ClutchSize  R : ClutchSize | 0.36 (0.15, 0.58)  0.11 (0.01, 0.21)  0.02 (–0.12, 0.15)  0.02 (–0.12, 0.17) | 0.39 (0.13, 0.64)  0.11 (0.01, 0.21)  0.03 (–0.11, 0.16)  0.03 (–0.11, 0.18) | 0.32 (0.12, 0.51)  0.12 (0.02, 0.22)  0.01 (–0.11, 0.13)  0.03 (–0.10, 0.17) | 0.35 (0.12, 0.59)  0.12 (0.02, 0.22)  0.04 (–0.08, 0.15)  0.06 (–0.07, 0.19) |
| Treatment (E = enlarged, R = reduced)  * Broods per year (centred and standardized)  *Intercept estimated for Treatment = E, and for mean broods per year, Separate slopes estimated for the effect of Broods per Year for each Treatment level* | Intercept  Treatment(R)  E : BroodsPerYear  R : BroodsPerYear | 0.34 (0.14, 0.55)  0.12 (0.02, 0.22)  –0.06 (–0.21, 0.09)  0.04 (–0.11, 0.18) | 0.37 (0.14, 0.61)  0.12 (0.02, 0.22)  –0.04 (–0.19, 0.12)  0.06 (–0.10, 0.21) | 0.32 (0.14, 0.50)  0.12 (0.03, 0.22)  –0.02 (–0.14, 0.11)  0.07 (–0.05, 0.20) | 0.35 (0.12, 0.57)  0.12 (0.03, 0.22)  0.00 (–0.13, 0.14)  0.09 (–0.04, 0.23) |
| Treatment (E = enlarged, R = reduced)  * Lifetime productivity (LP, centred and standardized)  *Intercept estimated for Treatment = E, and for mean lifetime productivity, Separate slopes estimated for the effect of LP for each Treatment level* | Intercept  Treatment(R)  E: LP  R : LP | 0.38 (0.18, 0.58)  0.12 (0.02, 0.22)  0.03 (–0.11, 0.17)  0.08 (–0.05, 0.22) | 0.40 (0.18, 0.62)  0.12 (0.02, 0.22)  0.03 (–0.10, 0.17)  0.08 (–0.05, 0.22) | 0.35 (0.19, 0.51)  0.12 (0.02, 0.22)  0.05 (–0.06, 0.17)  0.10 (–0.01, 0.22) | 0.37 (0.21, 0.53)  0.12 (0.02, 0.22)  0.06 (–0.05, 0.17)  0.11 (0.00, 0.22) |

# Table S8. The 10 highest sign-adjusted Hedges’ g values (G_flip) and associated variance (vi) from our data set in order from highest to lowest (1 = highest, 10 = lowest of the 10), with Study ID (i.e. unique study identifier) and Study.

| **Order** | **Study ID** | **Study** | **G_flip** | **vi** |
| --- | --- | --- | --- | --- |
| 1 | 60 | Ardia (2007) | 6.17 | 0.70 |
| 2 | 60 | Ardia (2007) | 5.85 | 0.64 |
| 3 | 60 | Ardia (2007) | 4.95 | 0.49 |
| 4 | 1003 | Komdeur *et al*. (2002) | 4.43 | 2.30 |
| 5 | 1003 | Komdeur *et al*. (2002) | 4.18 | 2.12 |
| 6 | 1003 | Komdeur *et al*. (2002) | 3.66 | 1.78 |
| 7 | 1894 | Smith *et al*. (1988) | 2.85 | 0.39 |
| 8 | 1003 | Komdeur *et al*. (2002) | 2.54 | 1.20 |
| 9 | 60 | Ardia (2007) | 2.36 | 0.21 |
| 10 | 2108 | Verhulst & Tinbergen (1997) | 2.20 | 0.29 |

# Fig. S1. Data visualizations to assess whether important methodological moderators were confounded with treatment or life-history variables. A–F illustrate the distribution of estimates from enlarged or reduced *versus* control treatments relative to each methodological moderator: (A) treatment duration (short = within a day, long = across days); (B) study design (within-subject or among-subject); (C) treatment stage (eggs or nestlings); (D) parental sex (male, female, or both); (E) response variable category (visit rates, feeding rates or delivery rates); and (F) relative manipulation size (number eggs/nestlings added/removed relative to control brood size). G–N illustrate the distribution of estimates for the two methodological moderators found to be important, response variable category (G–J), and relative manipulation size (K–N) as a function of the life-history variables clutch size (G and K), adult survival (H and L), broods per year (I and M), and lifetime productivity (J and N).

# Fig. S2. Graphical model of optimal parental care when benefit and cost curves are strongly non-linear (nearly step functions), which leads to threshold effects on care decisions. Condition-dependent defensive care in fish is a good example. The *x*-axis refers to parental effort towards current reproduction. The vertical dashed line illustrates the optimal level of parental care/desertion threshold. This is the parental investment level in current reproduction that maximizes total reproduction (i.e. current + future). To the left of this line, the benefit of increasing care to current offspring is greater than the cost to RRV (residual reproductive value), while to the right of this line, the benefit of increasing care to current offspring is less than the cost to RRV. Manipulations assessing this hypothesis would need to understand the biology of these curves to be effective.

# Supplemental references

Aho, T. (1997). *Determinants of breeding performance of the Eurasian treecreeper*. PhD thesis, University of Jyväskylä.

Allen, J. M., Hodinka, B. L., Barbera, R. A., Desjardin, G. C. & Williams, T. D. (2023). Variation in oxidative status, but not structural and physiological development, associated with changing ontogenetic environments. *Journal of Experimental Biology* **226**, jeb245974.

Arct, A., Drobniak, S. M., Dubiec, A., Martyka, R., Sudyka, J., Gustafsson, L. & Cichoń, M. (2022). The interactive effect of ambient temperature and brood size manipulation on nestling body mass in blue tits: an exploratory analysis of a long-term study. *Frontiers in Zoology* **19**(1), 9.

Ardia, D. R. (2007). Site- and sex-level differences in adult feeding behaviour and its consequences to offspring quality in tree swallows (*Tachycineta bicolor*) following brood-size manipulation. *Canadian Journal of Zoology* **85**(8), 847-854.

Arnold, T. W. (1993). Fledging success in experimentally manipulated broods of house wrens. *Wilson Bulletin* **105**(3), 448-454.

Arnold, T. W. (2011). An experimental study of fledging success in American Coots (*Fulica americana*): effects of brood size, food availablity, and hatching asynchrony. *Auk* **128**(4), 737-745.

Beissinger, S. R. (1990). Experimental brood manipulations and the monoparental threshold in snail kites. *American Naturalist* **136**(1), 20-38.

Bennett, P. M. & Owens, I. P. F. (2002). *Evolutionary ecology of birds: Life histories, mating systems and extinction*. Oxford University Press, Oxford, UK.

Billerman, S. M., Keeney, B. K., Rodewald, P. G. & Schulenberg, T. S. E. (2022). *Birds of the World*. Cornell Lab of Ornithology, Ithaca, NY, USA.

Boonekamp, J. J., Bauch, C. & Verhulst, S. (2020). Experimentally increased brood size accelerates actuarial senescence and increases subsequent reproductive effort in a wild bird population. *J Anim Ecol* **89**(6), 1395-1407.

Boonekamp, J. J., Salomons, M., Bouwhuis, S., Dijkstra, C. & Verhulst, S. (2014). Reproductive effort accelerates actuarial senescence in wild birds: an experimental study. *Ecology Letters* **17**(5), 599-605.

Bowers, E. K., Nietz, D., Thompson, C. F. & Sakaluk, S. K. (2014). Parental provisioning in house wrens: effects of varying brood size and consequences for offspring. *Behavioral Ecology* **25**(6), 1485-1493.

Brown, M. B. & Brown, C. R. (2020). Barn swallow (*Hirundo rustica*), version 1.0. In *Birds of the World*. (ed P. G. Rodewald). Cornell Lab of Ornithology, Ithaca, NY, USA.

BTO (British Trust for Ornithology) (2023). *BirdFacts: profiles of birds occurring in the United Kingdom*. British Trust for Ornithology, Thetford, UK.

Bulit, F. & Massoni, V. (2011). Apparent survival and return rate of breeders in the southern temperate White-rumped Swallow *Tachycineta leucorrhoa*. *Ibis* **153**(1), 190-194.

Butler, R. G., Buckley, D. E., Nettleship, D. N., Boesman, P. F. D. & Garcia, E. (2020). Black Guillemot (*Ceppus grille*), version 1.0. In *Birds of the World*. (ed S. M. Billerman). Cornell Lab of Ornithology, Ithaca, NY, USA.

Cauchard, L., Angers, B., Boogert, N. J., Lenarth, M., Bize, P., Doligez, B. & ine. (2017). An experimental test of a causal link between problem-solving performance and reproductive success in wild great tits. *Frontiers in Ecology and Evolution* **5**.

Chantler, P., Bonan, A. & Boesman, P. F. D. (2020). Pallid swift (*Apus pallidus*), version 1.0. In *Birds of the World*. (eds J. del Hoyo, A. Elliott, J. Sargatal, D. A. Christie and E. de Juana). Cornell Lab of Ornithology, Ithaca, NY, USA.

Collar, N., de Juana, E., Christie, D. A. & Boesman, P. F. D. (2020). Red-flanked bluetail (*Tarsiger cyanurus*), version 1.1. In *Birds of the World*. (eds J. del Hoyo, A. Elliott, J. Sargatal, D. A. Christie, E. de Juana and M. G. Smith). Cornell Lab of Ornithology, Ithaca, NY, USA.

Conrad, K. F. & Robertson, R. J. (1993). Patterns of parental provisioning by Eastern phoebes. *Condor* **95**(1), 57-62.

Cornelius Ruhs, E., Vézina, F., Walker, M. A. & Karasov, W. H. (2020). Who pays the bill? The effects of altered brood size on parental and nestling physiology. *Journal of Ornithology* **161**(1), 275-288.

Cornell Lab of Ornithology (2025). All About Birds. Cornell Lab of Ornithology, Ithaca, New York. https://www.allaboutbirds.org

Costanzo, A., Parolini, M., Bazzi, G., Khoriauli, L., Santagostino, M., Possenti, C. D., Romano, A., Nergadze, S. G., Rubolini, D., Giulotto, E. & Saino, N. (2016). Brood size, telomere length, and parent-offspring color signaling in barn swallows. *Behavioral Ecology* **28**(1), 204-211.

Cucco, M. & Malacarne, G. (1995). Increase of parental effort in experimentally enlarged broods of pallid-swifts. *Canadian Journal of Zoology* **73**(8), 1387-1395.

Dawson, R. D. (1999). *Causes and consequences of reproductive effort in American kestrels (Falco sparverius)*. PhD thesis, University of Saskatchewan.

Dykstra, C. R. & Karasov, W. H. (1993). Nesting energetics of house wrens (*Troglodytes aedon*) in relation to maximal rates of energy flow. *Auk* **110**(3), 481-491.

Edge, K. A., Jamieson, I. G. & Darby, J. T. (1999). Parental investment and the management of an endangered penguin. *Biological Conservation* **88**(3), 367-378.

Emaresi, G., Bize, P., Altwegg, R., Henry, I., van den Brink, V., Gasparini, J. & Roulin, A. (2014). Melanin-specific life-history strategies. *American Naturalist* **183**(2), 269-280.

Engelbretsen, S. I. & Slagsvold, T. (2023). An experimental study of trade-offs between brood size and moult in the blue tit (*Cyanistes caeruleus*). *Ibis* **165**, 781-789.

Ferretti, V., Llambias, P. E. & Martin, T. E. (2005). Life-history variation of a neotropical thrush challenges food limitation theory. *Proceedings of the Royal Society B-Biological Sciences* **272**(1564), 769-773.

Fokkema, R. W., Ubels, R. & Tinbergen, J. M. (2016). Great tits trade off future competitive advantage for current reproduction. *Behavioral Ecology* **27**(6), 1656-1664.

Furness, R. W., Boesman, P. F. D. & Garcia, E. (2020). Great skua (*Stercorarius skua*), version 1.0. In *Birds of the World*. (eds J. del Hoyo, A. Elliott, J. Sargatal, D. A. Christie and E. de Juana). Cornell Lab of Ornithology, Ithaca, NY, USA.

Galbraith, H. (1983). The diet and feeding ecology of breeding kittwakes (*Rissa tridactyla*). *Bird Study* **30**, 109-120.

Garcia-Campa, J., Müller, W., Rodriguez-Juncá, A. & Morales, J. (2023). When parents play favorites: brood demand shapes parental preference for offpsring UV color. *Behavioral Ecology* **34**(4), 700-707.

Gebczynski, A., Taylor, J. R. E. & Konarzewski, M. (1996). Growth of Dovekie (*Alle alle*) chicks under conditions of increased food demand at the nest: Two field experiments. *Canadian Journal of Zoology* **74**(6), 1076-1083.

González-Medina, E., Castillo-Guerrero, J. A. & Mellink, E. (2010). Parental and chick responses of Laughing Gulls (*Leucophaeus atricilla*) to increase of flight costs and brood size. *Journal of Ornithology* **151**(1), 219-225.

Gori, D. F. (1988). Adjustmnent of parental investment with mate quality by male yellow-headed backbirds (*Xanthocephalus xanthocephalus*). *Auk* **105**(4), 672-680.

Gow, E. (2014). *Parental care in northern flickers: sex-related patterns of foraging, provisioning, and habitat use*, University of Saskatchewan.

Gow, E. A., Wiebe, K. L. & Musgrove, A. (2015). Nest sanitation in response to short- and long-term changes of brood size: males clean more in a sex-role-reversed species. *Animal Behaviour* **104**, 137-143.

Green, D. J. & Ydenberg, R. C. (1994). Energetic expenditure of male ospreys provisioning natural and manipulateed broods. *Ardea* **82**(2), 249-262.

Gustafsson, L. & Nilsson, S. G. (1985). Clutch size and breeding success of pied and collared flycatchers *Ficedula* spp. in nest-boxes of different sizes. *Ibis* **127**, 380-385.

Gustafsson, L., Qvarnstrom, A. & Sheldon, B. C. (1995). Trade-offs between life-history traits and a secondary sexual character in male collared flycatchers. *Nature* **375**(6529), 311-313.

Hainstock, M. H., Smith, M. C., Carr, J. & Shutler, D. (2010). Parental investment and brood value in tree swallows, *Tachycineta bicolor*. *Behaviour* **147**(4), 441-464.

Heaney, V. (1997). *Within brood trade-offs in reproductive effort: An experimental study on the common tern: Sterna hirundo*, University of Glasgow.

Heaney, V. & Monaghan, P. (1995). A within-clutch trade-off between egg-production and rearing in birds. *Proceedings of the Royal Society B-Biological Sciences* **261**(1362), 361-365.

Hegner, R. E. & Wingfield, J. C. (1987). Effects of brood-size manipulations on parental investment, breeding success, and reproductive endocrinology of house sparrows. *Auk* **104**(3), 470-480.

Hinde, C. A. & Kilner, R. M. (2006). Negotiations within the family over the supply of parental care. *Proceedings of the Royal Society B-Biological Sciences* **274**(1606), 53-60.

Ibáñez-Álamo, J. D., Arco, L. & Soler, M. (2012). Experimental evidence for a predation cost of begging using active nests and real chicks. *Journal of Ornithology* **153**(3), 801-807.

Jelinek, V., Pozgayova, M., Honza, M. & Prochazka, P. (2016). Nest as an extended phenotype signal of female quality in the great reed warbler. *Journal of Avian Biology* **47**(3), 428-437.

Jones, G. (1987). Parent-offsring resource-allocation in swallows during nestling rearing: an experimental study. *Ardea* **75**(2), 145-168.

Klemp, S. (2000). Effects of parental effort on second brood, moult and survival in the Grey Wagtail *Motacilla cinerea*. *Ardea* **88**(1), 91-98.

Komdeur, J., Wiersma, P. & Magrath, M. (2002). Paternal care and male mate-attraction effort in the European starling is adjusted to clutch size. Proceedings of the Royal Society B-Biological Sciences 269(1497), 1253-1261.

Leach, A. G., Sedinger, J. S., Riecke, T. V., Van Dellen, A. W., Ward, D. H. & Boyd, W. S. (2019). Brood size affects future reproduction in a long-lived bird with precocial young. *American Naturalist* **193**(3), 458-471.

Leffelaar, D. & Robertson, R. J. (1986). Equality of feeding roles and the maintenance of monogamy in tree swallows. *Behavioral Ecology and Sociobiology* **18**(3), 199-206.

Leonard, M. L., Horn, A. G., Gozna, A. & Ramen, S. (2000). Brood size and begging intensity in nestling birds. *Behavioral Ecology* **11**(2), 196-201.

Lifjeld, J. T. & Slagsvold, T. (1991). Sexual conflict among polygynous pied flycatchers feeding young. *Behavioral Ecology* **2**(2), 106-115.

Lowther, P. E. & Cink, C. L. (2020). House sparrow (*Passer domesticus*), version 1.0. In *Birds of the World*. (ed S. M. Billerman). Cornell Lab of Ornithology, Ithaca, NY, USA.

Lozano, G. A. & Lemon, R. E. (1998). Parental-care responses by yellow warblers (*Dendroica petechia*) to simultaneous manipulations of food abundance and brood size. *Canadian Journal of Zoology-Revue Canadienne De Zoologie* **76**(5), 916-924.

Lucass, C., Korsten, P., Eens, M. & Muller, W. (2016). Within-family parent-offspring co-adaptation in a wild bird: on static traits, behavioural reaction norms, and sex differences. *Functional Ecology* **30**(2), 274-282.

Mariette, M. M. & Griffith, S. C. (2015). The adaptive significance of provisioning and foraging coordination between breeding partners. *American Naturalist* **185**(2), 270-280.

Martins, T. L. F. (1997). Fledging in the common swift, *Apus apus*: Weight-watching with a difference. *Animal Behaviour* **54**, 99-108.

Martins, T. L. F. & Wright, J. (1993*a*). Brood reduction in response to manipulated brood sizes in the common swift (*Apus apus*). *Behavioral Ecology and Sociobiology* **32**(1), 61-70.

Martins, T. L. F. & Wright, J. (1993*b*). Cost of reproduction and allocation of food between parent and young in the swift (*Apus apus*). *Behavioral Ecology* **4**(3), 213-223.

Massoni, V. & Reboreda, J. (2006). Breeding biology of the White‐rumped Swallow *Tachycineta leucorrhoa* in Buenos Aires Province, Argentina. *Ibis* **149**, 10-17.

McKinnon, R. A., Lagisz, M., Sridharah, S. Westneat, D. F., & Mathot, K. J. (2026). Data for: Trade-offs in avian parental care: a review of theory and meta-analysis of brood size manipulations. <https://doi.org/10.17605/OSF.IO/7KYF2>.

Moreno, J. (1987). Parental care in the wheatear (*Oenanthe oenanthe*) - effects of nestling age and brood size. *Ornis Scandinavica* **18**(4), 291-301.

Murphy, M. T., Armbrecth, B., Vlamis, E. & Pierce, A. (2000). Is reproduction by Tree Swallows cost free? *Auk* **117**(4), 902-912.

Murphy, M. T. & Pyle, P. (2020). Eastern kingbird (*Tyrannus tyrannus*), version 1.0. In *Birds of the World*. (ed P. G. Rodewald). Cornell Lab of Ornithology, Ithaca, NY, USA.

Musgrove, A. (2014). *Parental effort in the Northern Flicker (Colaptes auratus) and the trade-off between quantity and quality of offspring*. MSc thesis, University of Saskatchewan.

Nur, N. (1984). Feeding frequencies of nestling blue tits (*Parus caeruleus*): costs, benefits and a model of optimal feeding frequency. *Oecologia* **65**(1), 125-137.

O'Dea, R. E., Lagisz, M., Jennions, M. D., Koricheva, J., Noble, D. W. A., Parker, T. H., Gurevitch, J., Page, M. J., Stewart, G., Moher, D. & Nakagawa, S. (2021). Preferred reporting items for systematic reviews and meta-analyses in ecology and evolutionary biology: a PRISMA extension. *Biological Reviews* **96**(5), 1695-1722.

Olsen, J. & Tucker, A. D. (2003). A brood-size manipulation experiment with Peregrine Falcons, *Falco peregrinus*, near Canberra. *Emu* **103**(2), 127-132.

Ostreiher, R. & Heifetz, A. (2015). The blessing of having younger nestmates: the case of the Arabian babbler. *Behavioral Ecology* **27**(2), 393-400.

Patterson, C. B., Erckmann, W. J. & Orians, G. H. (1980). An experimental study of parental investment and polygyny in male blackbirds. *American Naturalist* **116**(6), 757-769.

Pollet, I. L., Shutler, D., Chardine, J. W. & Ryder, J. P. (2020). Ring-billed gull (*Laru delawarensis*), version 1.0. In *Birds of the World*. (ed A. F. Poole). Cornell Lab of Ornithology, Ithaca, NY, USA.

Pryke, S. R. & Griffith, S. C. (2009). Socially mediated trade-offs between aggression and parental effort in competing color morphs. *American Naturalist* **174**(4), 455-464.

Przybylo, R. & Merilä, J. (2000). Intersexual niche differentiation in the blue tit (*Parus caeruleus*). *Biological Journal of the Linnean Society* **69**(2), 233-244.

Raymond, C. T. I. (2020). Western cattle-egret (*Ardea ibis*), version 1.1. In *Birds of the World*. (eds P. G. Rodewald, B. K. Keeney, S. M. Billerma and M. A. Bridwell). Cornell Lab of Ornithology, Ithaca, NY, USA.

Ridenour, M. (2020). *Insulin-like growth factor 1 and the hormonal mediation of sibling rivalry in eastern bluebirds (Sialia sialis)*. MSc thesis, Oklahoma State University.

Roulin, A., Ducrest, A. L. & Dijkstra, C. (1999). Effect of brood size manipulations on parents and offspring in the barn owl *Tyto alba*. *Ardea* **87**(1), 91-100.

Rytkonen, S. (1995). Parental care in the willow tit (*Parus montanus*): Nest defence and nestling provisioning in the light of the parental investment theory. *Acta Universitatis Ouluensis Series A Scientiae Rerum Naturalium* (265), 1-37.

Saino, N., Ambrosini, R., Rubolini, D., Romano, M., Caprioli, M., Romano, A. & Parolini, M. (2018). Carry-over effects of brood size on morphology, reproduction, and lifespan in barn swallows. *Behavioral Ecology and Sociobiology* **72**(2), 30.

Saino, N., Calza, S. & Moller, A. P. (1997). Immunocompetence of nestling barn swallows in relation to brood size and parental effort. *Journal of Animal Ecology* **66**(6), 827-836.

Saino, N., Ninni, P., Incagli, M., Calza, S., Sacchi, R. & Moller, A. P. (2000). Begging and parental care in relation to offspring need and condition in the barn swallow (*Hirundo rustica*). *American Naturalist* **156**(6), 637-649.

Schreiber, B. A. & Schreiber, R. W. (2020). Red-tailed tropicbird (*Phaethon rubricauda*), version 1.0. In *Birds of the World*. (ed S. M. Billerman). Cornell Lab of Ornithology, Ithaca, NY, USA.

Sendecka, J., Cichon, M. & Gustafsson, L. (2007). Age-dependent reproductive costs and the role of breeding skills in the Collared flycatcher. *Acta Zoologica* **88**(2), 95-100.

Shields, M. (2020). Brown pelican (*Palecanus occidentalis*), version 1.0. In *Birds of the World*. (ed A. F. Poole). Cornell Lab of Ornithology, Ithaca, NY, USA.

Shutler, D., Clark, R. G., Fehr, C. & Diamond, A. W. (2006). Time and recruitment costs as currencies in manipulation studies on the costs of reproduction. *Ecology* **87**(11), 2938-2946.

Siefferman, L. & Hill, G. E. (2005). Male eastern bluebirds trade future ornamentation for current reproductive investment. *Biology Letters* **1**(2), 208-211.

Siefferman, L. & Hill, G. E. (2007). The effect of rearing environment on blue structural coloration of eastern bluebirds (*Sialia sialis*). *Behavioral Ecology and Sociobiology* **61**(12), 1839-1846.

Siikamaki, P., Haimi, J., Hovi, M. & Ratti, O. (1998). Properties of food loads delivered to nestlings in the pied flycatcher: effects of clutch size manipulation, year, and sex. *Oecologia* **115**(4), 579-585.

Simmons, R. (1986). Food provisioning, nestling growth and experimental manipulation of brood size in the african redbreasted sparrowhawk *Accipiter rufiventris*. *Ornis Scandinavica* **17**(1), 31-40.

Slagsvold, T. & Rohwer, S. (2000). Sexual conflict in birds with biparental care: Should a parent desert a brood with low reproductive value? *Ornis Norvegica* **23**(1), 38-49.

Smallwood, J. A. & Bird, D. M. (2020). American Kestrels (*Falco sparverius*), version 1.0. In *Birds of the Wolrd*. (eds A. F. Poole and F. B. Gill). Cornell Lab of Ornithology, Ithaca, NY, USA.

Smith, H. G., Käller, H., Fontell, K. & Ljungström, M. (1988). Feeding frequency and parental division of labour in the double-brooded great tit *Parus major* - Effects of manipulating brood size. *Behavioral Ecology and Sociobiology* **22**(6), 447-453.

Sousa, N. O. d. M. (2008). *Teste de fatores que afetam o tamanho da ninhada de Elaenia chiriquensis (Tyrannidae) no Cerrado do Brasil Central*. Dissertação de Mes-trado, thesis, Universidade de Brasília.

Sousa, N. O. M. & Marini, M. A. (2013). A negative trade-off between current reproductive effort and reproductive success: an experiment with clutch-size in a tropical bird. *Emu-Austral Ornithology* **113**(1), 8-18.

Styrsky, J. N., Brawn, J. D. & Robinson, S. K. (2005). Juvenile mortality increases with clutch size in a neotropical bird. *Ecology* **86**(12), 3238-3244.

Sudyka, J., Casasole, G., Rutkowska, J. & Cichoń, M. (2016). Elevated reproduction does not affect telomere dynamics and oxidative stress. *Behav Ecol Sociobiol* **70**(12), 2223-2233.

Thomson, R. L., Griesser, M., Laaksonen, T. & Korpimäki, E. (2014). Brood size manipulations in a spatially and temporally varying environment: male Tengmalm’s owls pass increased reproductive costs to offspring. *Oecologia* **176**(2), 423-430.

Turner, A. (2020). Fairy martin (*Petrochelidon ariel*), version 1.0. In *Birds of the World*. (eds J. del Hoyo, A. Elliott, J. Sargatal, D. A. Christie and E. de Juana). Cornell Lab of Ornithology, Ithaca, NY, USA.

Verhulst, S. & Tinbergen, J. M. (1997). Clutch size and parental effort in the Great Tit *Parus major*. *Ardea* **85**(1), 111-126.

Ursino, C. A. (2016). *Efecto de dos parásitos de cría sobre el cuidado parental de una especie con cría cooperativa, el Músico (Agelaioides badius) ; Effects of two brood parasites on the parental behavior of a cooperatively breeding host, the Baywing (Agelaioides badius)*, Universidad de Buenos Aires. Facultad de Ciencias Exactas y Naturales.

Weeks Jr., H. P. (2020). Eastern phoebe (*Sayornis phoebe*), version 1.0. In *Birds of the World*. (ed A. F. Poole). Cornell Lab of Ornithology, Ithaca, NY, USA.

Westneat, D. F., Mutzel, A., Bonner, S. & Wright, J. (2017). Experimental manipulation of brood size affects several levels of phenotypic variance in offspring and parent pied flycatchers. *Behavioral Ecology and Sociobiology* **71**(6).

Wheelwright, N. T. & Rising, J. D. (2020). Savannah sparrow (*Passerculus sandwichensis*), version 1.0. In *Birds of the World*. (ed A. F. Poole). Cornell Lab of Ornithology, Ithaca, NY, USA.

Whittingham, L. A. (1989). An experimental study of paternal behavior in red-winged blackbirds. *Behavioral Ecology and Sociobiology* **25**(1), 73-80.

Wiebe, K. L. & Moore, W. S. (2020). Northern flicker (*Calaptes auratus*), version 2.1. In *Birds of the World*. (eds P. G. Rodewald and B. K. Keeney). Cornell Lab of Ornithology, Ithaca, NY, USA.

Wiersma, P. & Tinbergen, J. M. (2003) No nocturnal energetic savings in response to hard work in free-living great tits. *Netherlands Journal of Zoology* **52**(2), 263-279.

Winkler, D. W., Hallinger, K. K., Ardia, D. R., Robertson, R. J., Stutchbury, B. J. & Cohen, R. R. (2020). Tree swallow (*Tachycineta bicolor*), version 1.0. In *Birds of the Wolrd*. (ed A. F. Poole). Cornell Lab of Ornithology, Ithaca, NY, USA.

Wright, J., Both, C., Cotton, P. A. & Bryant, D. (1998). Quality vs. quantity: energetic and nutritional trade-offs in parental provisioning strategies. *Journal of Animal Ecology* **67**(4), 620-634.

Wright, J. & Cuthill, I. (1990). Biparental care: short-term manipulation of partner contribution and brood size in the starling, *Sturnus vulgaris*. *Behavioral Ecology* **1**(2), 116-124.

Wright, J., Hinde, C., Fazey, I. & Both, C. (2003). Begging signals more than just short-term need: cryptic effects of brood size in the pied flycatcher (Ficedula hypoleuca). *Behavioral Ecology and Sociobiology* **52**(1), 74-83.

Yasukawa, K. & Searcy, W. A. (2020). Red-winged blackbird (*Agelaius phoeniceus*), version 1.0. In *Birds of the World*. (ed P. G. Rodewald). Cornell Lab of Ornithology, Ithaca, NY, USA.
